# Airborne microbial biodiversity and seasonality in Northern and Southern Sweden

**DOI:** 10.7717/peerj.8424

**Published:** 2020-01-27

**Authors:** Edvin Karlsson, Anna-Mia Johansson, Jon Ahlinder, Moa J. Lundkvist, Navinder J. Singh, Tomas Brodin, Mats Forsman, Per Stenberg

**Affiliations:** 1 Department of Molecular Biology, Umeå University, Umeå, Sweden; 2 Department of Biological Agents, Division of CBRN Defense and Security, Swedish Defense Research Agency, Umeå, Sweden; 3 Department of Wildlife, Fish, and Environmental Studies, Swedish University of Agricultural Sciences, Umeå, Sweden; 4 Department of Ecology and Environmental Sciences, Umeå University, Umeå, Sweden

**Keywords:** Airborne biodiversity, Microbial seasonality, High-throughput sequencing, Metabarcoding, eDNA

## Abstract

Microorganisms are essential constituents of ecosystems. To improve our understanding of how various factors shape microbial diversity and composition in nature it is important to study how microorganisms vary in space and time. Factors shaping microbial communities in ground level air have been surveyed in a limited number of studies, indicating that geographic location, season and local climate influence the microbial communities. However, few have surveyed more than one location, at high latitude or continuously over more than a year. We surveyed the airborne microbial communities over two full consecutive years in Kiruna, in the Arctic boreal zone, and Ljungbyhed, in the Southern nemoral zone of Sweden, by using a unique collection of archived air filters. We mapped both geographic and seasonal differences in bacterial and fungal communities and evaluated environmental factors that may contribute to these differences and found that location, season and weather influence the airborne communities. Location had stronger influence on the bacterial community composition compared to season, while location and season had equal influence on the fungal community composition. However, the airborne bacterial and fungal diversity showed overall the same trend over the seasons, regardless of location, with a peak during the warmer parts of the year, except for the fungal seasonal trend in Ljungbyhed, which fluctuated more within season. Interestingly, the diversity and evenness of the airborne communities were generally lower in Ljungbyhed. In addition, both bacterial and fungal communities varied significantly within and between locations, where orders like Rhizobiales, Rhodospirillales and Agaricales dominated in Kiruna, whereas Bacillales, Clostridiales and Sordariales dominated in Ljungbyhed. These differences are a likely reflection of the landscape surrounding the sampling sites where the landscape in Ljungbyhed is more homogenous and predominantly characterized by artificial and agricultural surroundings. Our results further indicate that local landscape, as well as seasonal variation, shapes microbial communities in air.

## Introduction

Spatial and temporal variation influence the composition and abundance of microorganisms in the environment (Bardgett et al., 1999; De Vries et al., 2012; Tedersoo et al., 2014; Van der Gucht et al., 2007). Microorganisms adapting to these variations have allowed multiple species to coexist over time, resulting in species having different abundancies over seasonal cycles; exemplified in soil where seasonal fluctuations in available resources influence fungal and bacterial community composition (Bardgett et al., 2005; Bardgett et al., 1999). Studying spatial and temporal differences are important to improve our understanding how various factors shape the diversity and composition of microorganisms in different environments (Ladau & Eloe-Fadrosh, 2019).

Despite being essential constituents of ecosystems, it is not until the last decade or so that spatial and temporal dynamics of microorganisms have been assessed in larger scale, due to challenges in cultivating and taxonomically classifying microorganisms from environmental samples. However, with the advances in high throughput DNA sequencing, environmental DNA (eDNA) isolated from a wide number of sources, e.g., soil, water and air, has emerged as a promising method to study spatial and temporal dynamics in microbial communities (Bowers et al., 2013; Chen et al., 2018; Ji et al., 2013; Taberlet et al., 2012; Thomsen & Willerslev, 2015).

Using air as a source is especially attractive, since air provides a more comprehensive view of the total microbial diversity compared to many other single sample types, as it contains microorganisms of both terrestrial and aquatic origin, from many different sources such as soil, lakes, oceans, and plant surfaces (Bowers et al., 2013; Caliz et al., 2018; Cao et al., 2014; Smets et al., 2016). However, studying how local environmental and temporal variation affect airborne microbial communities is challenging. The abundance of taxa in air is not only affected by the abundance of microorganisms in the local environment, but also by the means and ease which these microorganisms disperse through air, as exemplified by different dispersal mechanisms among fungi (Elbert et al., 2007). Some microorganisms exist in air primarily for reproductive reasons during a limited time of the year, while others exist in air through more stochastic processes. In addition, microorganisms may disperse over very long distances (Barberan et al., 2015; Smith et al., 2013), which may further complicate the analysis of local effects.

Nevertheless, several studies do indicate that local sources contribute to the microbial composition in ground level air (Bowers et al., 2011; Docherty et al., 2018; Gandolfi et al., 2015; Mhuireach et al., 2019; Peay & Bruns, 2014; Tignat-Perrier et al., 2019) and that season, as well as temporal shifts in weather, influence the composition of airborne communities (Bowers et al., 2013; Franzetti et al., 2011; Gandolfi et al., 2015; Innocente et al., 2017; Yamamoto et al., 2012; Zhen et al., 2017). This suggests that airborne communities may be useful to study factors that shape the diversity and composition of microorganisms in the local environment.

Few studies have, however, performed continuous measurements of the microbial composition in ground level air over more than one year or one location. In addition, there are only a few studies of airborne communities at high latitude (Cuthbertson et al., 2017; Santl-Temkiv et al., 2018). Studying microbial response to environmental fluctuations at high latitude, such as the Arctic where seasonal differences are large, is promising for identifying factors important for temporally structuring the microbial community. Especially, since the larger contrast between the seasons may have a stronger influence on the ecosystem (Lisovski, Hoye & Klaassen, 2017).

Our goal was to examine spatial and temporal differences in airborne microbial communities over two full consecutive years, in the Arctic boreal (Kiruna) and the southern nemoral zone (Ljungbyhed) of Sweden, and evaluate whether local factors contributed to these differences. For this purpose, we used a unique source of archived air filters collected within the Swedish national radioactive fallout monitoring program. We first evaluated the usefulness of the archived air filters for studying temporal change in airborne communities by performing plant (*rbcL*) metabarcoding analyses and validated our sequence data with pollen counts. We then performed bacterial (16S-V4) and fungal (ITS) metabarcoding analyses to assess differences in microbial communities across location and season, and compared these differences to differences in local land cover and weather.

## Materials and Methods

### Air sampling

The air samples used in this study have been collected within the framework of the Swedish radioactive fallout monitoring program (Söderström et al., 2007; Söderström, Ban & Lindh, 2008), which has collected and archived air samples over more than five decades. Ground level air was filtered at a rate of ∼1,000 m^3^/h through 60 ×60 cm glass fiber filters (Type CS 5.0, Camfil, Trosa, Sweden). The filters were changed twice a week and combined into a weekly sample by compressing $ \frac{3}{4} $ of the two filters into a cylinder shape (13 mm, ø60 mm), which was then archived in a sealed plastic container at room temperature. For the current study, we chose 52 air filters each from Kiruna (67.84°N, 20.42°E) and Ljungbyhed (56.08°N, 13.22°E) representing every second week during the years 2006 and 2007 (*n* = 104). These two years were selected based on being the two consecutive years that differed most in terms of weather between 1999 and 2014. This was defined as the largest Euclidean distance between two consecutive years when combining the monthly averages of temperature, precipitation, atmospheric pressure and relative humidity at both locations. Two blank filters were also compressed and used as negative controls. Prior to DNA extraction, the air filters were randomized and coded (Table S1).

### DNA extraction

The DNA extraction was performed in eight batches using a custom MoBio Powerwater kit (Mobio Laboratories, San Diego, CA, USA) (Table S1). Three replicate pieces were punched out of each compressed, cylinder shaped, filter using a biopsy punch (Ø8 mm, Integra Miltex, Plainsboro, NJ, USA. Each punch represents ∼1% of the total filter volume) and placed individually in two mL tubes containing 1.0 g of 0.1 mm and 0.5 g of 1.0 mm zirconia/silica beads (BioSpec, Bartlesville, OK, USA). A volume of one mL preheated (55 °C) PW1 solution (Mobio Laboratories) was added to each tube and incubated for 10 min at 65 °C in a water bath. The tubes were then agitated in a FastPrep-24 instrument (MP Biomedicals, Santa Ana, CA, USA) for 30s at 5.5 m/s, followed by centrifugation at 16, 000 ×g for 15 min. The supernatant was recovered from each tube (0.5 mL) and put on ice. Another 0.5 mL PW1 solution was added and the beat beating and centrifugation was repeated to recover a second volume of supernatant. This was repeated again to yield a total of three supernatants per filter piece (the last centrifugation was shortened to 5 min). The repeated bead beating protocol was adopted from Wunderlin et al. (2013). The DNA was then isolated according to the manufacturer’s instructions, with the exception that the three supernatants originating from the same filter piece were pooled and loaded onto the same spin filter and the volume of the binding buffer was changed accordingly. The resulting three DNA samples from each filter were pooled and further concentrated using DNA Clean & Concentrator-5 (Zymo Research, Irvine, CA, USA) into 75 µl elution buffer according to the manufacturer’s instructions.

### Amplicon generation

Amplicons targeting *rbcL* (plants), ITS (fungi) and 16S-V4 (bacteria) were generated by PCR according to the 16S Metagenomic Sequencing Library Preparation guide (15044223B, Illumina, San Diego, CA, USA) with a few modifications. The amplicon reaction contained 2.5 µl DNA, 200 nM reverse and forward primers (Table S2) in a total of 25 µl 1 × 5PRIME HotMasterMix (QuantaBio, Beverly, MA, USA). The PCR was performed at 94 °C for 3 min followed by 30 cycles of 94 °C for 45s, 50 °C for 60s and 72 °C for 90s, and with a final elongation at 72 °C for 10 min (Walters et al., 2016). Five µl of purified amplicons was then indexed by using 100 nM forward and reverse indexing primers (Nextera XT indexing kit v2 set A, Illumina) per reaction, in a total of 50 µl 1 × 5PRIME HotMasterMix (QuantaBio) and cycled five times using the same cycling conditions as described above. The concentration of the final indexed amplicons was measured by Qubit Fluorometric Quantification (Invitrogen, Carlsbad, CA, USA) using the Qubit dsDNA HS Assay kit.

### Sequencing

The amplicons were pooled and sequenced on four separate MiSeq (Illumina) sequencing runs according to Table S1. The pooled amplicons were spiked with 5% PhiX (Illumina) and sequenced using the MiSeq reagent kit v3 (paired end, 2 ×250/300, Table S1). The raw sequence data is available at EBI (http://www.ebi.ac.uk/ena), accession number PRJEB23947.

### Sequence processing and OTU picking

The obtained reads were trimmed and filtered using Trimmomatic (v0.32) (Bolger, Lohse & Usadel, 2014) with default settings. The minimum read length was set to 100 bp for *rbcL* and 150 bp for ITS and 16S-V4. The paired reads were then merged using Flash (v1.2.11) (Magoc & Salzberg, 2011) with a minimum overlap of 10 bp and a maximum overlap of 250 bp for *rbcL* and 300 bp for ITS and 16S-V4. Primer sequences were removed by Cutadapt (v1.13) (Martin, 2011) using a maximum error rate of 0.2. For *rbcL*, sequences outside the length range 108–112 bp were discarded.

The processed amplicon sequences were then clustered into operational taxonomic units (OTUs) using Qiime (Quantitative Insights Into Microbial Ecology) (v.1.9.1) (Caporaso et al., 2010). BOLD systems (2017-03-29) (Ratnasingham & Hebert, 2007), UNITE (ver7_97_01.08.2015) (Koljalg et al., 2005) and Greengenes (release 8.15.13) (DeSantis et al., 2006) were used as reference sequence databases for *rbcL*, ITS and 16S-V4, respectively. The *rbcL* reference database was constructed by downloading all *rbcL* sequences and associated taxonomy from boldsystems.org and converted into the appropriate format using a custom python script. The *rbcL* reference sequences were then clustered into operational taxonomic units (OTUs) using UCLUST (Edgar, 2010) at 99% sequence similarity with default settings in QIIME (pick_otus.py parameters: enable_rev_strand_match = True). The script pick_rep_set.py in QIIME was then used to pick a representative sequence for each OTU. Taxonomy was assigned to each representative sequence using the script assign_taxonomy.py. The representative sequences and their taxonomy were used as the final *rbcL* reference sequence database.

The amplicon sequences were clustered into OTUs using pick_open_reference_otus.py in QIIME. Prior to OTU picking the sequences were pre-filtered to remove sequences that had lower than 60% sequence identity to the reference database. Open reference OTU picking was then performed at 99% sequence identity for *rbcL* and 97% for ITS and 16S-V4 using the following pick_otus.py parameters: enable_rev_strand_match = True, max_accepts = 20, max_rejects = 500, stepwords = 20, word_length = 12, similarity = 0.97 (ITS, 16S-V4)/ 0.99 (*rbcL*) and assign_taxonomy.py parameters: assignment_method = uclust (16S-V4, *rbcL*)/ blast (ITS). The resulting OTU tables were filtered to remove OTUs containing less than 0.00005% of the total number of sequences, unassigned OTUs and 16S-V4 OTUs assigned to chloroplast and mitochondria. In addition, if the maximum OTU sequence count of the blanks exceeded 5% of the maximum sequence count of a given location (Kiruna or Ljungbyhed) the OTU sequence count was set to zero for that location.

### Normalization

The samples were normalized by rarefication i.e., subsampled to an equal sequence depth. Rarefaction plots were generated from the OTU tables using alpha_rarefaction.py in QIIME. The rarefaction level for each marker was chosen by eye based on the plateauing of the number of observed OTUs (16S-V4: 8,970 sequences, ITS: 22,278 sequences, *rbcL*: 36,277 sequences) (Fig. S1).

### Plant sequence and pollen count comparison

To evaluate how accurately the air filters have stored information on temporal abundance we compared our plant sequence data to daily pollen counts from Abisko (68.35°N, 18.83°E). Pollen counts were obtained from the Palynological Laboratory at the Swedish Museum of Natural History (NRM). Biweekly Kiruna (67.84°N, 20.42°E) sequence counts of the order Fagales were compared to the summed daily pollen counts (count per m^3^ air) of *Alnus*, *Betula*, *Carpinus*, *Corylus*, *Quercus* and *Fagus*, the Pinales sequence counts to the pollen counts of *Picea*, *Pinus* and *Juniperus*, the Ericales sequence counts to the pollen counts of Ericaceae, the Poales sequence counts to the pollen counts of Cyperaceae, Poaceae, Juncaceae and *Typha* pollen, the Malpaghiales sequence counts to the pollen counts of *Salix* and *Populus* and the Rosales sequence counts to the pollen counts of *Filipendula*, *Urtica* and *Ulmus*. The OTU table were in this comparison normalized by the amplicon concentration to provide a quantitative (opposed to relative) estimate of the sequence abundance prior to library pooling. The quantitative OTU sequence abundance estimate was calculated by multiplying each OTU sequence count with the calculated normalization coefficient for each sample (Table S1).

### Land cover data

We compared differences in local land cover at the air filtering sites to assess whether these differences could explain geographic differences in microbial composition. Land cover data (Svenska Marktäckedata – NMD Hagner et al. (2005)), with a resolution of 25 × 25 m (dating back to 2003) was obtained and updated to match the years of the filter data. Spatial data was obtained from the Swedish Forest Agency for all clear-felled areas since 2000, as this is the most frequent land cover change in Sweden. Forest habitats that had been clear-felled were reclassified and these were also aged when necessary into younger forest (0-5 years in general for southern regions and 0-10 years for the northern study area - due to lower productivity in this region). The habitat map contain 60 habitat classes (see http://mdp.vic-metria.nu/miljodataportalen/ for original habitat classes). From this data, the proportion of different land cover types were retrieved within one, five and 10 km radius from each air filter station.

### Source tracking analysis

To evaluate if any bacteria observed in air originated from a potential fecal source related to the surrounding landscape we performed source tracking on the bacterial OTUs. Fecal source predictions were made using SourceTracker, version 1.0.0 (Knights et al., 2011) as described in Hagglund et al. (2018). OTUs that were based on *de novo* clustered sequences were excluded from the analysis to match the source tracking library. The source tracking library included OTUs generated from 16S-V4 amplicon sequences from calf, cow, domestic bird, dog, horse, pig, sheep and wild bird feces, as well as sewage (Hagglund et al., 2018). The rarefying depth in the source tracking analysis was set to 8,970 to reduce the required computational time, which is similar to the recommendations provided by Ahmed et al. (2015).

### Weather data

To categorize the samples into defined seasons (spring, summer, autumn, winter) and evaluate relationships between microbial diversity and meteorological variables we used weather data retrieved from the Swedish Meteorological and Hydrological Institute (SMHI). The weather data was based on daily gridded data with a resolution of 4 × 4 km. SMHI’s definition for the meteorological seasons was used to categorize the weeks by season, where spring starts on the first day of seven consecutive days above 0 °C and below 10 °C, summer starts on the first day of five consecutive days above 10 °C, autumn starts on the first day of five consecutive days below 10 °C and winter starts on the first day of five consecutive days below 0 °C. In addition, spring cannot start earlier than 15th of February and autumn cannot start earlier than 1st of August.

### Statistical analysis

Statistical analysis was performed in the statistical program R (R Core Team, 2013). To assess the correlation with plant sequence and pollen counts we calculated a general nonlinear correlation estimate by using the R package nlcor (Ranjan & Najari, 2019), after concatenating all plant order data sets used for comparison into a single series.

Relationships between the plant, bacterial and fungal communities were evaluated using Non-metric Multidimensional Scaling (NMDS) based on Bray-Curtis dissimilarity as implemented in the R package vegan (Oksanen et al., 2019). Bray-Curtis dissimilarities were calculated on Hellinger transformed rarefied OTU tables. The package ggplot2 (Wickham, 2016) was used for visualization and the goeveg function ordiselect (Goral & Schellenberg, 2018) was used to select the most abundant OTUs for the OTU score plots.

PERMANOVA and the Adonis package in vegan was used to partition the variance explained by location and season (Oksanen et al., 2019). Dispersion homogeneity was also evaluated using the Betadisper package in vegan to test if the groups had the same dispersion (distance to centroid). The *p*-values for Adonis and Betadisper were calculated using 999 permutations.

Differential abundance testing was performed using Wilcoxon rank-sum test to evaluate significant geographic differences between bacterial and fungal orders. The *p*-values were adjusted using the Benjamini–Hochberg method to adjust for multiple testing. Wilcoxon rank test was also used to test if the distribution of order and OTU richness among the samples was significantly different between locations and seasons (wilcox.test and pairwise.wilcox.test function in R).

To assess geographic differences in land cover within one, five and 10 km radius of the two air filter stations the land cover diversity was estimated by calculating the Shannon entropy (*H′*) (Shannon, 1948):

\begin{eqnarray*}{H}^{{}^{{^{\prime}}}}=-\sum _{i=1}{p}_{i}\log \nolimits 2 \left( {p}_{i} \right) \end{eqnarray*}

where *p*_*i*_ is the proportion of land cover type.

Spearman’s rank correlations was performed to find linear or monotonic relationships between the abundance of different taxa, as well as relationships between OTU/order richness and weather parameters (cor.test function in R).

To assess temporal trends in OTU and order richness, local regression (LOESS) lines were fitted to the observations using ggplot2 (geom_smooth, span = 0.5)(Wickham, 2016).

## Results and Discussion

### Sequencing

After removing OTUs with low sequence counts in the filter station samples and OTUs with high sequence counts in the blank samples, the median sequence count ± median absolute deviation for the bacterial (16S-V4), fungal (ITS) and plant (*rbcL*) amplicon markers were 55,814 ± 37,677, 119,533 ± 73,446 and 70,919 ± 70,064, respectively. Subsequent normalization by rarefication resulted in a total of *n* = 94 bacterial samples, *n* = 86 fungal samples and *n* = 58 plant samples (Fig. S1). Over the two locations and the two years studied, we observed a large number of taxa (230 bacterial, 136 fungal and 84 plant orders) (Table S3, File S1), suggesting that the air filters have stored information on a large diversity of organisms.

### Plant abundance shows expected variation

To assess how well the air filters have stored information on temporal abundance of airborne communities, we first performed plant specific amplicon sequencing. Our results show that the sequence counts are in most cases highly consistent and correlated with the corresponding pollen counts (Fig. S2) (non-linear correlation estimate = 0.60, *p* = 0.01). Minor deviations between the sequence and pollen counts might be explained by the inability of DNA based techniques to distinguish between pollen and other plant materials. In addition, we compare sequence counts assigned at order level, which can include more species than the ones included in the pollen data. For example, the sequence counts of the order Malpighiales are compared to *Salix* and *Populus* pollen counts where most of the sequences in our study do not have higher taxonomic resolution than order. Nevertheless, given that the two data sets are generated separately in time and space with two independent methods, the high degree of correlation is striking.

Ordination by NMDS also shows that samples collected in similar weeks also had similar plant composition, due to orders that are known to be present in air within those weeks (Fig. S3). This is exemplified by Pinales that bloom and release their pollen during weeks in spring and summer. In addition, high sequence abundance of Lycopodiales and Bryophytes like Polytrichales, Splachnales, Hypnales and Dicranales explained the clustering of weeks in early spring and autumn in Kiruna, which is consistent with the dispersal of aerial propagules among species within these orders (Manninen et al., 2014; Ross-Davis & Frego, 2004). Similarly, the abundance of Equisetales coincided with expected spore release period; from spring to end of summer depending on species (Magee & Ahles, 2007). Also, the presence of Myrtales in the end of the summer can be explained by species that disperse their seeds by wind at this time of the year, e.g., *Epilobium* (Myerscough, 1980).

Thus, together with the pollen count comparison, it is clear that the air filters have maintained information regarding temporal abundance, despite years of storage.

### Geographic location contribute to differences in microbial composition

Bacterial and fungal metabarcoding analyses showed that the orders observed in Kiruna and Ljungbyhed are generally consistent with communities typically found in air, although the proportions differ in some cases (Bowers et al., 2013; Bowers et al., 2011; Cuthbertson et al., 2017; Frohlich-Nowoisky et al., 2009; Yamamoto et al., 2012).

A large proportion of the airborne microbial communities were made up by a small number of orders. This was especially apparent among bacterial orders in Ljungbyhed (Figs. S5, S6), where Clostridiales and Bacillales contributed to 38.8% and 35.9% of the total sequence count, respectively. In comparison, Clostridiales and Bacillales only contributed to 2.7% and 6.6% of the sequences in Kiruna. Instead, the majority of sequences in Kiruna were assigned to Rhizobiales (24.4%), Rhodospirillales (20.4%) and Acidobacteriales (11.0%). However, despite these proportional differences, the most common bacterial orders found in Kiruna and Ljungbyhed were, with only a few exceptions, the same across the two locations (Fig. S5).

Compared to bacteria, a slightly larger number of fungal orders contributed to the majority of sequences (Fig. S5). Still, orders like Sordariales (26.4%) in Ljungbyhed and Agaricales (12.1%) in Kiruna contributed to a large proportion of the sequences. Among the most abundant orders we also found Polyporales (6.0%), Lecanorales (5.2%), Pleosporales (5.1%) and Taphrinales (4.5%) in Kiruna and Russulales (9.4%), Capnodiales (8.4%), Polyporales (5.7%) and Pleosporales (4.1%) in Ljungbyhed.

**Figure 1:**
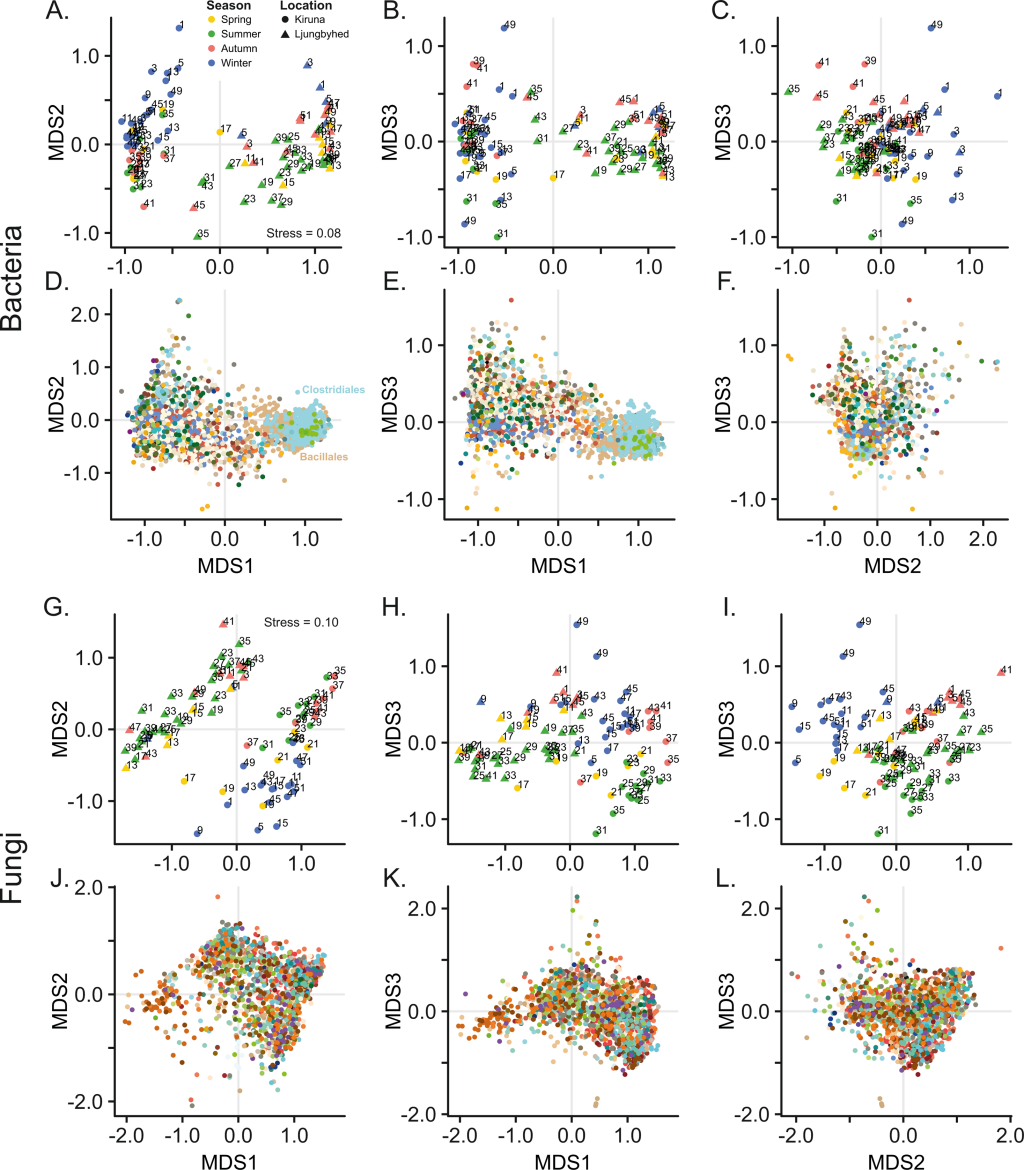
Non-metric multidimensional scaling (NMDS) of bacterial and fungal community composition. Sample score plots shown in three dimensions for (A–C) bacteria and (G–I) fungi based on Bray–Curtis dissimilarities of Hellinger transformed sequence counts. The samples are labelled by week number. The corresponding (D–F) bacterial and (J–L) fungal OTU scores for the three dimensions are colored according to taxonomic identity (order). For clarity, only the 20% most abundant OTUs are displayed. Color keys are shown in Fig. S4.

Ordination by NMDS further shows that bacterial and fungal communities are clearly separated by location (Fig. 1). Location explained 26.1% and 15.3% of the observed variation among the bacterial and fungal communities, respectively (PERMANOVA, Bacteria; *F* = 34.9, *p* < 0.001, Fungi; *F* = 17.7, *p* < 0.001). However, we observed a difference in dispersion (distance to centroid) within location among the fungal communities (Betadisper, *F* = 7.28, *p* = 0.011), which also might contribute to the observed effect of location. This was not observed among the bacterial communities (Betadisper, *F* = 0.54, *p* = 0.468).

The geographic separation of the bacterial and fungal communities can be explained by differences in community diversity, where the diversity, in terms of evenness and richness was generally higher in Kiruna compared to Ljungbyhed (Figs. S6, S7). For instance, both the bacterial and fungal order richness, as well as the fungal OTU richness was higher in Kiruna (Fig. S7) (Bacterial order richness - Kiruna; Median = 49, IQR = 29, Ljungbyhed; Median = 38, IQR = 29, Wilcoxon rank-sum test *p* < 0.05, Fungal order richness - Kiruna; Median = 67, IQR = 22, Ljungbyhed; Median = 51, IQR = 45, *p* < 0.05, Fungal OTU richness - Kiruna; Median = 548, IQR = 764, Ljungbyhed; Median = 233, IQR = 615, *p* < 0.01). However, the bacterial OTU richness was an exception and was significantly higher in Ljungbyhed (Fig. S7) (Bacterial OTU richness - Kiruna; Median = 401, IQR = 370, Ljungbyhed; Median = 893, IQR = 536, *p* < 0.01), but a large proportion of these OTUs (37.4%) belonged to the two most common orders, Bacillales (18.1%) and Clostridiales (19.3%) (File S1).

In conjunction with the clear differences observed in diversity, compositional differences also explained the geographic separation between the communities. Among the bacterial orders, this is partially due to the high abundance of the bacterial orders Bacillales (35.9%) and Clostridiales (38.8%) in Ljungbyhed. As a result, most other orders were less abundant compared to Kiruna (Fig. S8). Along with Bacillales (Log_2_-ratio = 2.45, Wilcoxon rank-sum test, *p* < 0.001) and Clostridiales (Log_2_-ratio = 3.83, *p* < 0.001), Turicibacterales (Log_2_-ratio = 5.91, *p* < 0.001) and OPB54 (Log_2_-ratio = 4.00, *p* < 0.001) were differentially more abundant in Ljungbyhed (Fig. S8). These orders all belong to the class Firmicutes that are commonly associated with soil and mammalian gut (Rosenberg et al., 2014).

Among the large number of bacterial orders that were comparatively more abundant in Kiruna, most noticeable are Vibrionales (Log_2_-ratio = 7.15, *p* = 0.0029), Coriobacteriales (Log_2_-ratio = 4.71, *p* < 0.001) and Nostocales (Log_2_-ratio = 4.23, *p* = 0.005) (Fig. S8). Vibrionales is commonly associated with aquatic environments (Imhoff, 2005), while Nostocales, in addition to aquatic environments, may be linked to e.g., mosses and lichens (Rikkinen, 2007; Santi, Bogusz & Franche, 2013). In contrast, Coriobacteriales is more associated with mammalian body habitats (Clavel, Lepage & Cd, 2014).

The geographic differences were also evident among the fungal orders and similarly, there were only a few orders that were more abundant in Ljungbyhed; exemplified by Wallemiales (Log_2_-ratio = 10.7, *p* < 0.001) and Sordariales (Log_2_-ratio = 3.54, *p* < 0.001) - both found in e.g., soil (Klaubauf et al., 2010; Zalar et al., 2005) (Fig. S8). In Kiruna we observed comparatively higher abundance of Lecanoromycetes, containing lichen forming fungi (Kirk et al., 2008) and orders such as Acarosporales (Log_2_-ratio = 8.97, *p* < 0.001), Lecanorales (Log_2_-ratio = 2.63, *p* < 0.001) and Teloschistales (Log_2_-ratio = 3.78, *p* = 0.005). Moreover, Mucorales (Log_2_-ratio = 8.11, *p* < 0.001) associated with soil, plants, animals and fungi (Hoffmann et al., 2013), Venturiales (Log_2_-ratio = 5.54, *p* < 0.001) associated with pathogenicity in plants e.g., *Salix* (Shen, Zhang & Zhang, 2016) and Leotiales (Log_2_-ratio = 6.69, *p* < 0.001) associated with for example ericaceous soil and ericoid mycorrhiza (Bougoure et al., 2007; Kjoller, Olsrud & Michelsen, 2010) were all more abundant in Kiruna (Fig. S8).

In an attempt to explain the geographic differences in airborne microbial communities, we compared differences in the landscape surrounding the air filter stations. The land cover analysis confirmed that the landscape differed considerably between the stations, both in terms of land cover composition and land cover diversity (Fig. S9). Within one kilometer radius the landscape surrounding the station in Ljungbyhed consisted mainly of an airport (56.9%) and arable land (11.0%), with low presence of forests and other natural vegetation types. On the contrary, the landscape surrounding the filter station in Kiruna were more heterogeneous with many different forest types (37.5%), shrub and herbaceous vegetation (8.76%) and mires (6.36%). Due to this heterogeneity, the land cover types in Kiruna displayed a noticeably higher diversity (H′= 4.13) and evenness compared to Ljungbyhed (H′= 2.75) (Figs. S9A and S9B).

At five and 10 km radius the diversity and evenness of the land cover types became more similar (Fig. S9) (5 km; H′_Kiruna_ = 4.79, H′_Ljungbyhed_ = 4.35, 10 km; H′_Kiruna_ = 4.90, H′_Ljungbyhed_ = 4.30), but still with large differences in the land cover composition and a considerable agricultural component in Ljungbyhed in the form of arable land (5 km; 20.8%, 10 km = 15.8%) and pastures (5 km; 8.29%, 10 km = 6.04%) (Fig. S9).

Given that local sources contribute to the airborne microbial composition, the more heterogeneous landscape closest to the air filter station may provide one explanation to the generally more diverse airborne communities observed in Kiruna. Since the geographic differences in land cover diversity becomes more similar over a larger area, it may also suggest that a large part of the observed diversity originates from the land close to the air filter station. Indeed, although fungal spores and bacteria are capable of long distance dispersal, probabilistically, a large proportion will only travel a short distance from its source (Galante, Horton & Swaney, 2011; Vos & Velicer, 2008). In addition, the land cover types in Kiruna, may themselves harbor a larger species diversity as the surrounding area mainly consisted of natural vegetation types. The landscape close to the air filter station in Ljungbyhed were in contrast highly artificial, possibly having a negative impact on the species diversity. Thus, the lower diversity observed in Ljungbyhed might be reduced or even reversed if the filter station was positioned within a more heterogeneous or less artificial area.

The different land cover types may also explain the compositional differences in the airborne communities. In Kiruna, it is more expected that cyanobacteria (e.g., Nostocales), lichen-forming fungi (e.g., Lecanoromycetes) and plant associated fungal orders (e.g., Taphrinales, Venturiales and Leotiales) are more abundant where forests, shrubs, herbaceous vegetation and mires dominate the landscape. Similarly, it is not surprising that we observed a larger abundance of spore forming plants in Kiruna (e.g., Bryophyta, Equisetales and Lycopodiophyta).

Likewise, the high proportion of arable land that surrounded the air filter station in Ljungbyhed could explain the high abundance of Firmicutes (Bacillales and Clostridiales), potentially originating from soil and spread of organic fertilizers. Firmicutes can be highly abundant in soil (Kuramae et al., 2012). Firmicutes are also enriched in manure treated soil (Das et al., 2017), present in high levels in swine manure (Lim et al., 2018) and in airborne dust collected in pig stables (Vestergaard et al., 2018). In addition, we found a strong correlation between many families within Firmicutes and fungal families within Sordariales; found in both agricultural soils (Klaubauf et al., 2010) and manure (Gu et al., 2017) (Figs. S10,  S11), which further supports a link between the microbial composition and the agricultural landscape in Ljungbyhed.

To further reinforce this link, we evaluated if organic fertilizers could have contributed to the microbial communities in Ljungbyhed by investigating if any bacterial sequences were associated with a fecal source. Indeed, fecal source tracking showed that a small proportion (less than 2%) of the community in Ljungbyhed is predicted to originate from either a domestic bird or pig fecal source (Fig. S12). The proportion of fecal associated sequences increased in early spring (2006-w.13 and 2007-w.5) and declined again in late summer (2006-w.31 and 2007-w.35) (Fig. S12). Once more, this supports that the agricultural landscape contributed to the microbial composition in air.

It is, however, notable that we observed relatively low abundance of other soil related bacteria in Ljungbyhed e.g., Acidobacteria, Actinobacteria and Alphaproteobacteria (Lauber et al., 2009). This may be explained by differences in ease of dispersal, differential preservation of DNA on the filters or differential DNA extraction. Compared with many other DNA extraction protocols, our protocol is based on sequential bead beating, which in theory provides a better representation of the microbial composition as the protocol takes the different physical properties of different microorganisms into account. This approach improves yields of endospore-forming bacteria and should thus minimize bias in DNA extraction arising from inefficient lysis of endospores (Wunderlin et al., 2013).

### Airborne microbial diversity and composition fluctuates over seasons

In addition to the geographic differences observed in the microbial airborne communities there were also substantial temporal variation within and between the locations. NMDS show that both the bacterial and fungal communities are separated by seasonal belonging (Fig. 1). Season explained, however, more of the variation in the fungal communities, where 17.4% (PERMANOVA, *F* = 3.36, *p* < 0.001) of the variation was explained by season, compared to 9.72% (PERMANOVA, *F* = 2.17, *p* < 0.001) in the bacterial communities. This could not be explained by differences in dispersion (distance to centroid) among the seasonal groups (Betadisper, Bacteria; *F* = 0.35, *p* = 0.79, Fungi; *F* = 0.95, *p* = 0.41).

**Figure 2:**
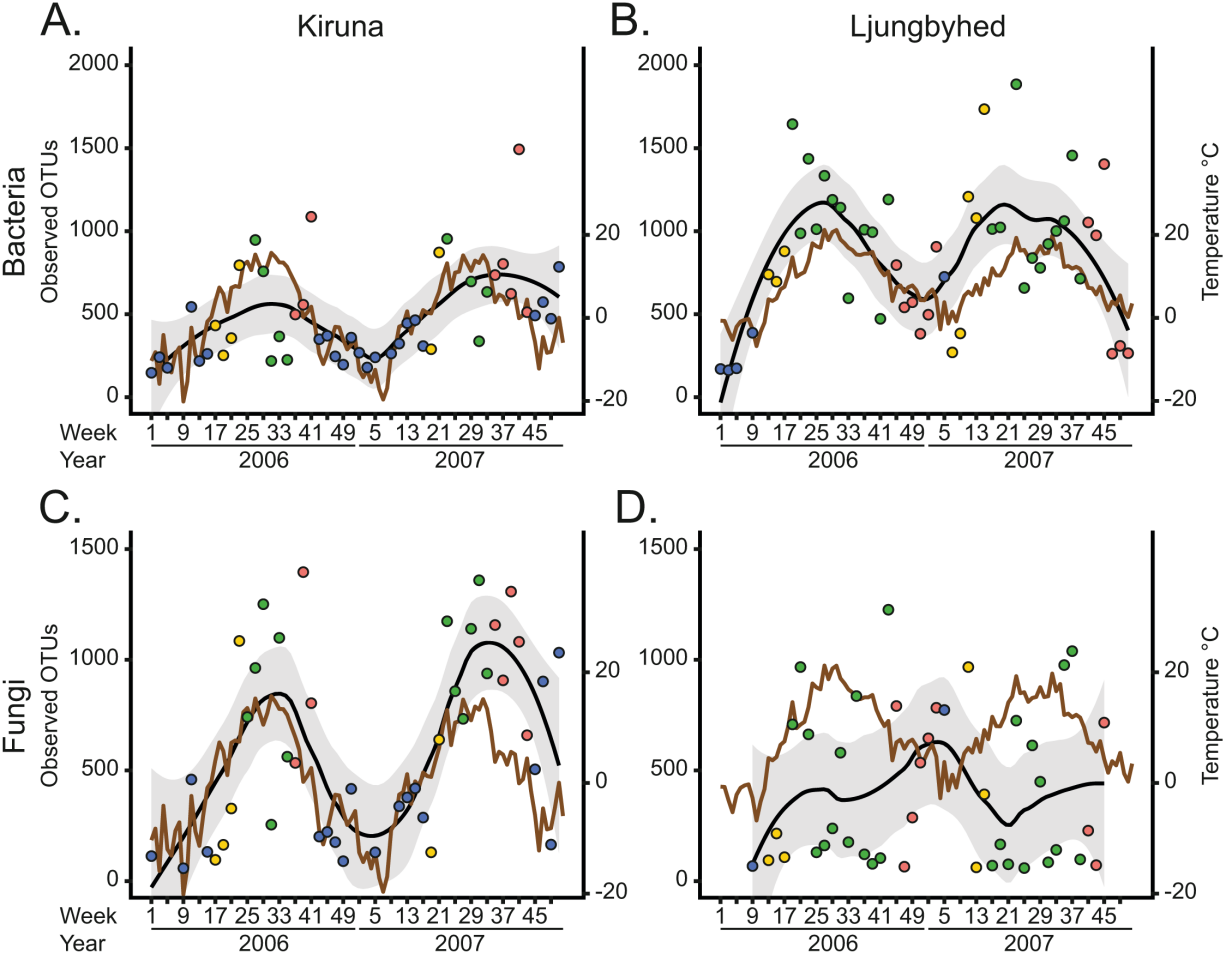
The observed number of OTUs over time and location. (A) Weekly bacterial OTU richness in Kiruna. (B) Weekly bacterial OTU richness in Ljungbyhed. (C) Weekly fungal OTU richness in Kiruna. (D) Weekly fungal OTU richness in Ljungbyhed. Weeks are colored by season (spring; yellow, summer; green, autumn; red and winter; blue). A local regression (LOESS) curve is fitted to the observations to display seasonal trends (black line). The standard error of the LOESS curve is depicted in grey. For comparison, the weekly average temperature is shown (brown line).

The seasonal differences are partially explained by seasonal fluctuations in diversity, which mostly followed a unimodal trend over the year (Fig. 2). The diversity, in terms of OTU richness, was generally highest during the spring, summer and autumn and lowest during the winter when the temperatures were low. Indeed, the bacterial and fungal OTU richness showed in most cases a positive linear or monotonic relationship with temperature (Spearman’s rank correlation, bacteria-Kiruna; *R*^2^ = 0.24, *p* < 0.001, bacteria-Ljungbyhed; *R*^2^ = 0.38, *p* < 0.001, fungi-Kiruna; *R*^2^ = 0.34, *p* < 0.001, (Fig. S13). The exception is the fungal communities in Ljungbyhed, where the diversity fluctuated more within season and showed no linear or monotonic relationship with temperature (fungi-Ljungbyhed; *R*^2^ = 0.002, *p* = 0.67). Similar trends and relationships with temperature were observed at order level, but at this taxonomic resolution they were less pronounced (Figs. S14, S15).

Besides the influence of season and temperature, we also found linear or monotonic relationships between bacterial community diversity and several other weather parameters (Figs. S13 , S14). The OTU richness in Ljungbyhed showed a strong negative correlation with humidity (*R*^2^ = 0.64, *p* < 0.001) and cloud cover (*R*^2^ = 0.54, *p* < 0.001) and a strong positive correlation with visibility (*R*^2^ = 0.69, *p* < 0.001). The bacterial communities in Kiruna showed a similar relationship, but compared to Ljungbyhed the correlations were weaker (humidity; *R*^2^ = 0.11, *p* < 0.05, cloud cover; *R*^2^ = 0.14, *p* < 0.05, visibility; *R*^2^ = 0.48, *p* < 0.001). These correlations are consistent with promoted aerial bacterial dispersal in dry weather (Tong & Lighthart, 2000). Further indicating that dry weather have had a stronger influence on shaping the airborne bacterial communities in Ljungbyhed, which may be linked to the arable land and high abundance of organisms related to soil.

**Figure 3:**
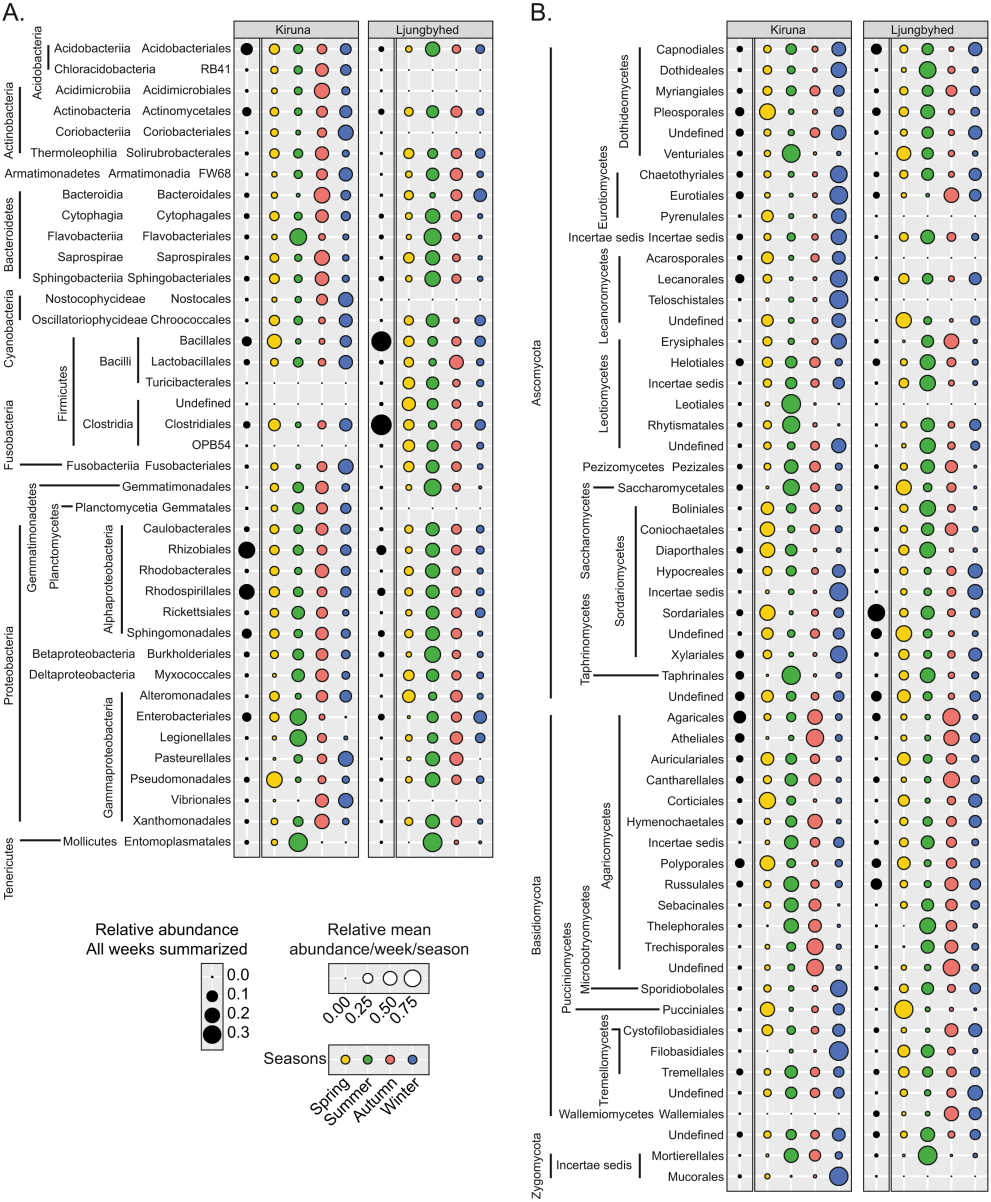
Observed order abundance across the sampling period and season. The relative order abundance across the sampling period (black circles) in Kiruna and Ljungbyhed, respectively, together with the average weekly relative abundance per season (colored circles) for (A) bacterial and (B) fungal orders. The seasonal abundance represents the total number of sequences observed in a given season divided by the number of weeks belonging to the season. Orders with a sequence abundance below 0.1% of the total sequence abundance are not shown.

The fungal diversity had overall weaker linear or monotonic relationships with the weather (Fig. S13). The fungal OTU richness in Kiruna was, in addition to temperature, only significantly correlated with visibility (*R*^2^ = 0.26, *p* < 0.001) and the fungal OTU richness in Ljungbyhed was only weakly positively correlation with precipitation (*R*^2^ = 0.10, *p* < 0.05), wind gust (*R*^2^ = 0.16, *p* < 0.05) and wind average (*R*^2^ = 0.16, *p* < 0.05). The weaker relationships observed could be related to the more distinct seasonality among fungal communities, as compared to bacterial communities. Thus, an overall weaker relationship with weather over the entire time period can be expected.

However, it should be noted that many of the weather parameters varies with season. Temperature showed a weak, but significant, positive linear or monotonic relationship with visibility (Kiruna; *R*^2^ = 0.20, *p* < 0.001, Ljungbyhed; *R*^2^ = 0.26, *p* < 0.001) and a negative linear or monotonic relationship with humidity (Kiruna; *R*^2^ = 0.14, *p* < 0.001, Ljungbyhed; *R*^2^ = 0.28, *p* < 0.001) and cloud cover (Only in Ljungbyhed; *R*^2^ = 0.24, *p* < 0.001), which makes it is hard to distinguish between direct and indirect relationships.

The temporal diversity trends of the fungal communities and their relationships with weather were strikingly different in Kiruna and Ljungbyhed (Figs. 2C and 2D, Fig. S13). The stronger association between diversity and temperature in Kiruna might be explained by the overall colder climate in Kiruna, characterized by a long period of snow coverage during winter, when growth and dispersal are more restricted. Conversely, in Ljungbyhed the temperatures rarely reached below freezing, potentially allowing fungal growth and dispersal during a larger part of the year. The clear temporal differences in diversity may also be related to differences in the landscape and the timing of host development and available nutrients (Chen et al., 2018; Nicolaisen et al., 2017). In Ljungbyhed where a relatively large proportion of arable land surrounds the air filter station, crops likely influence the fungal communities. It has been shown that airborne fungal diversity peak in spring and autumn in fields of growing crops (Chen et al., 2018), which could be one explanation to the sometimes low diversity observed during summer in Ljungbyhed. However, despite this sporadically low diversity observed in the summer, the fungal diversity generally peaked in either summer or autumn (Fig. 2D).

The summer in Ljungbyhed was typically associated with a peak in the relative abundance of fungal orders belonging to Ascomycota, while the autumn was more associated with a peak in Basidiomycota (Fig. 3). The orders of Basidiomycota had similar seasonal preferences in both locations. However, in contrast to Ljungbyhed, many orders of Ascomycota had higher relative abundance during the winter in Kiruna, similar to what has been observed at high altitude (Caliz et al., 2018) (Fig. 3). Since the landscape in Kiruna is covered in snow during winter the higher relative abundance of Ascomycetes may originate from more distant sources or come from fungi growing on trees above the snow surface, e.g., lichen forming Lecanoromycetes (Sonesson, Osborne & Sandberg, 1994). Nonetheless, there were exceptions to the winter preference among the Ascomycetes. Leotiomycetes, as well as Pezizales and Taphrinales were generally more abundant during summer in both locations (Fig. 3), where Taphrinales contain species associated with wooden plants like birch (Phillips & Burdekin, 1992).

The general preference for autumn among orders of Basidiomycota were related to a high abundance of Agaricomycetes that contain mushroom forming species, typically dispersing their spores during autumn (shown in other studies Frohlich-Nowoisky et al., 2009). Polyporales was one exception, which displayed a higher abundance in spring (Fig. 3). This is, however, consistent with the timing of sporulation of some species within the order (Schigel, Niemelä & Kinnunen, 2006). Similarly, Pucciniales showed a consistent spring preference (Fig. 3); an order containing many relevant plant pathogens, for example in spruce (Hietala, Solheim & Fossdal, 2008).

Among the bacterial orders, there were a relatively consistent seasonal preference for summer in Ljungbyhed (Wilcoxon rank-sum test, *p* < 0.05) (Fig. 3, Fig. S16). The seasonal preferences were less consistent in Kiruna, but there was an overall tendency for a higher preference for autumn or winter (Fig. 3, Fig. S16). The preference for autumn in Kiruna was partially related to orders belonging to one of the largest phyla, Proteobacteria, where most orders were more abundant in autumn, and summer to some extent. The seasonal preference of Proteobacteria was even stronger in Ljungbyhed, where all but Alteromonadales were most abundant in summer, but also here many of the same orders had elevated abundances in autumn.

Furthermore, many orders in Kiruna within Bacteroidetes were preferentially more abundant in autumn, with the exception of Flavobacteriales, which displayed a higher abundance during the summer in both locations; an order associated with soil and aquatic sources (McBride, 2014). Similarly, Entomoplasmatales was also more abundant in summer, which may be expected from an order related to arthropods and plants (Gasparich, 2014) (Fig. 3). Enterobacteriales had a similar seasonal preference in Kiruna (Fig. 3). In Ljungbyhed, however, the order occurred sporadically throughout summer, autumn and winter (Fig. 3).

The distinct seasonal preferences among the bacterial and fungal orders within and between locations, may be associated with geographic differences in environmental sources, climate and timing of available nutrients or hosts. Also, different species within individual orders may have different seasonal preferences. For example, in Kiruna most of the Russulales sequence counts are made up by the families Peniophoraceae, Russulaceae and Stereaceae while in Ljungbyhed Bondarzewiaceae makes up most of the sequence count (File S1).

In this study, we have characterized bacterial and fungal communities in ground level air in Northern and Southern Sweden. We have also tried to assess whether differences in these communities can be attributed to geographic differences in land cover, season or temporal change in weather. We acknowledge that not only the abundance of individual organisms in nature affects the observed abundance of taxa in the filters, but also how these organisms are dispersed in air. Geographic and temporal variation may not necessarily be related to seasonal changes within the local ecosystem, but may also be related to spatial or temporal variation in dispersal ability. In addition, differential DNA extraction efficiency among different organisms, storage, PCR bias and potential cross contamination may be confounding factors. Nevertheless, our results show that archived air filters are well-suited for studying airborne microorganisms and how they change over time. Although, these filters cannot resolve the absolute abundance of organisms in nature, the filters should accurately resolve long term trends in the relative abundance of individual groups of organisms.

The air filters used in this study represent a fraction of over 15,000 archived weekly air samples collected over five decades within Swedish radioactive fallout monitoring program. This archive constitute an extraordinary resource that likely provides one of the most extensive records of high-resolution historical biodiversity over the past decades in the world. We anticipate that the archive will provide insights into the influence of long term processes on biodiversity, such as climate and environmental change, data for modelling future trends in biodiversity, predictions of pathogen abundance and a way to monitor alien invasive species.

## Conclusions

We demonstrate that archived air filters intended for radioactive fallout measurements can be used to study spatial and temporal variation in airborne microbial communities, despite years of storage. In addition, we show that ground level air sampled in Kiruna, in Northern Sweden, generally contain a higher microbial diversity than in Ljungbyhed, in Southern Sweden. Moreover, the airborne bacterial and fungal diversity show overall the same pattern over the seasons, irrespective of location, with a peak during the warmer parts of the year, except for fungal seasonal pattern in Ljungbyhed, which fluctuate more within season. Both bacterial and fungal community composition vary significantly within and between location, due to geographic and seasonal differences, where the agricultural landscape in Ljungbyhed had a visible effect on the bacterial and fungal communities. We extend the current knowledge of spatial and temporal variation among airborne microbial communities and our results further indicate that local landscape, as well as seasonal variation, shapes microbial communities in air.

##  Supplemental Information

10.7717/peerj.8424/supp-1 Table S1 Amplicon and sequencing information

10.7717/peerj.8424/supp-2 Supplemental Information 1 Supplemental Figures and Tables

10.7717/peerj.8424/supp-3 File S1 OTU tables

## Additional Information and Declarations

### Data Availability

The following information was supplied regarding data availability: Sequence data is available at the European Nucleotide Archive: PRJEB23947.
