# Supplementary material for: Airborne microbial biodiversity and seasonality in Northern and Southern Sweden
Source: PeerJ. 2020 Jan 27;8:e8424. doi: 10.7717/peerj.8424 (PMC6991134; doi:10.7717/peerj.8424)
Supplement: Supplemental Information 1 [file peerj-08-8424-s002.pdf]

Airborne microbial biodiversity and seasonality in Northern and Southern Sweden.

Edvin Karlsson[1,2*], Anna-Mia Johansson[1], Jon Ahlinder[2], Moa J. Lundkvist[1], Navinder J. Singh[3], Tomas Brodin[3], Mats Forsman[2], Per Stenberg[2,4*]

1. Department of Molecular Biology, Umeå University, Umeå, Sweden
2. CBRN Defense and Security, Swedish Defense Research Agency (FOI), Umeå, Sweden
3. Department of Wildlife, Fish, and Environmental Studies, SLU, Umeå
4. Department of Ecology and Environmental Sciences, Umeå University, Umeå, Sweden

*Correspondence to per.stenberg@umu.se, edvin.karlsson@umu.se

Supplementary information

Table S2. Amplicon primer sequences used for generating amplicons for 16S-V4, ITS and *rbcL*, respectively. Gene-specific sequence and sequences associated with the references are emboldened.

Marker	Primer sequence (5'-3')	Ref.
16S-V4-515f	TCGTCGGCAGCGTCAGATGTGTATAAGAGACAG**GTGYCAGCMGCCGCGGTAA**	1, 2
16S-V4-806r	GTCTCGTGGGCTCGGAGATGTGTATAAGAGACAG**GGACTACNVGGGTWTCTAAT**	1, 3
ITS1f	TCGTCGGCAGCGTCAGATGTGTATAAGAGACAG**CTTGGTCATTTAGAGGAAGTAA**	1, 4
ITS2	GTCTCGTGGGCTCGGAGATGTGTATAAGAGACAG**GCTGCGTTCTTCATCGATGC**	1, 5
rbcL Z1aF	TCGTCGGCAGCGTCAGATGTGTATAAGAGACAG**ATGTCACCACCAACAGAGACTAAAGC**	6
rbcL 19bR	GTCTCGTGGGCTCGGAGATGTGTATAAGAGACAG**CTTCTTCAGGTGGAACTCCAG**	6

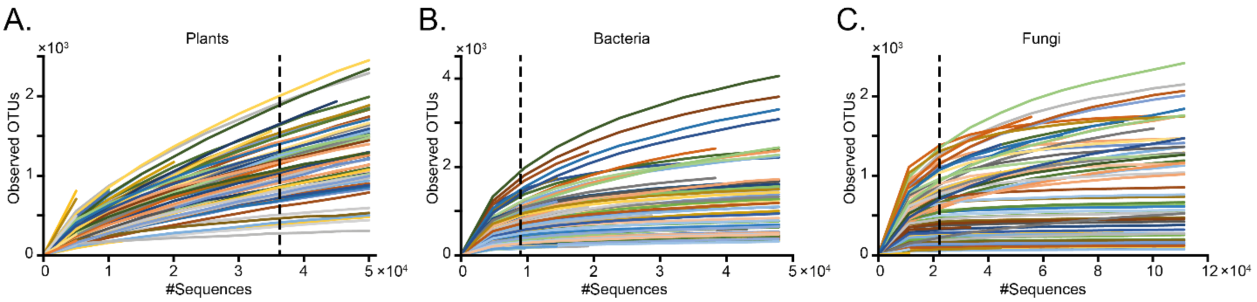

Figure S1. Rarefaction plots for **(A)** plants (*rbcL*), **(B)** bacteria (16S-V4) and **(C)** fungi (ITS) showing the number of OTUs for each sequence subsampling level. Each sample is subsampled from ten sequences up to the median sequence count, in ten steps. The rarefaction level chosen for the principal component analysis is shown by a dashed line (16S-V4: 8970 sequences, ITS: 22278 sequences, *rbcL*: 36277 sequences).

Table S3. Number of observed orders, families and genera over the two locations and two years studied.

Taxonomic group (marker)	Orders	Families	Genera
Plants (*rbcL*)	84	236	382
Bacteria/Archaea (16S-V4)	230	452	980
Fungi (ITS)	136	356	1107
**Total**	450	1046	2471

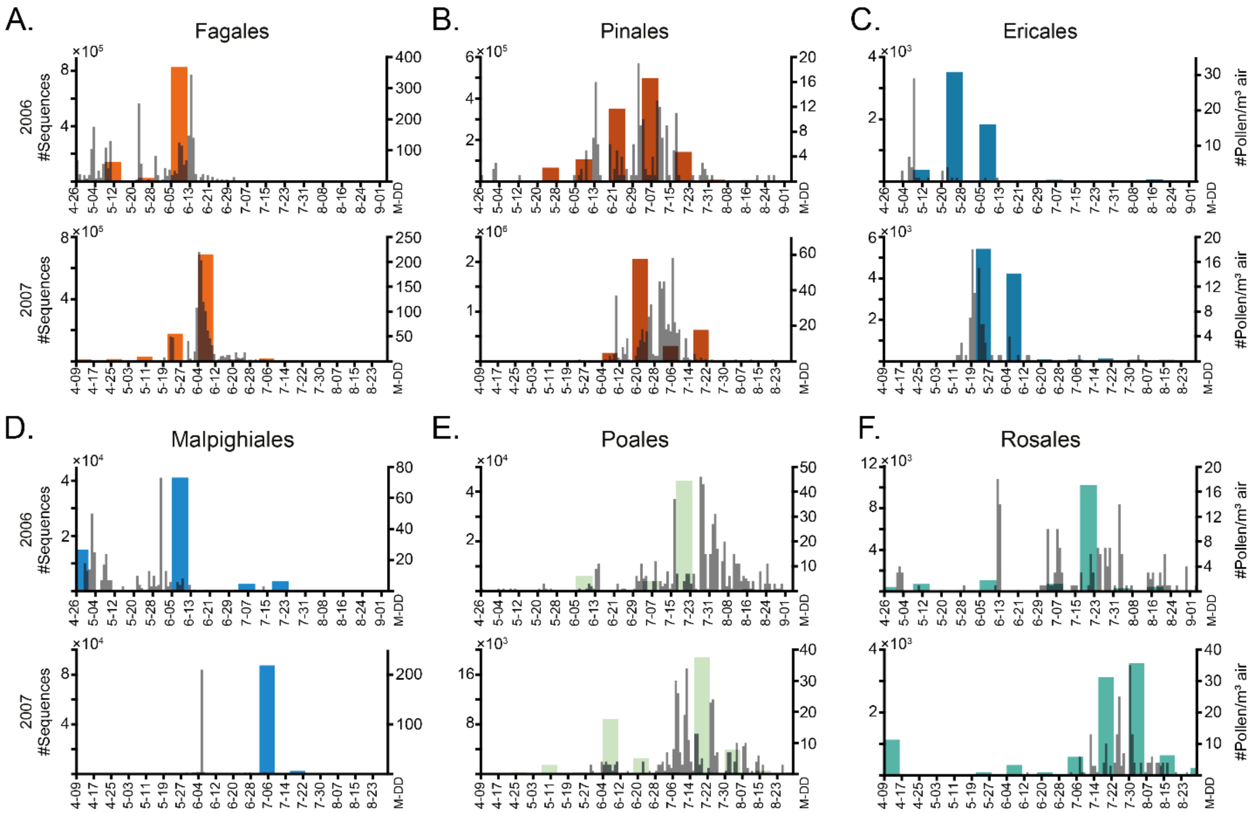

Figure S2. Comparison between the biweekly distributions of sequences assigned to **(A)** Fagales, **(B)** Pinales, **(C)** Ericales, **(D)** Malpighiales, **(E)** Poales and **(F)** Rosales with daily pollen counts (counts/m^3, grey) from Abisko, in the years 2006 and 2007. The time period shown is restricted to the pollen sampling period.

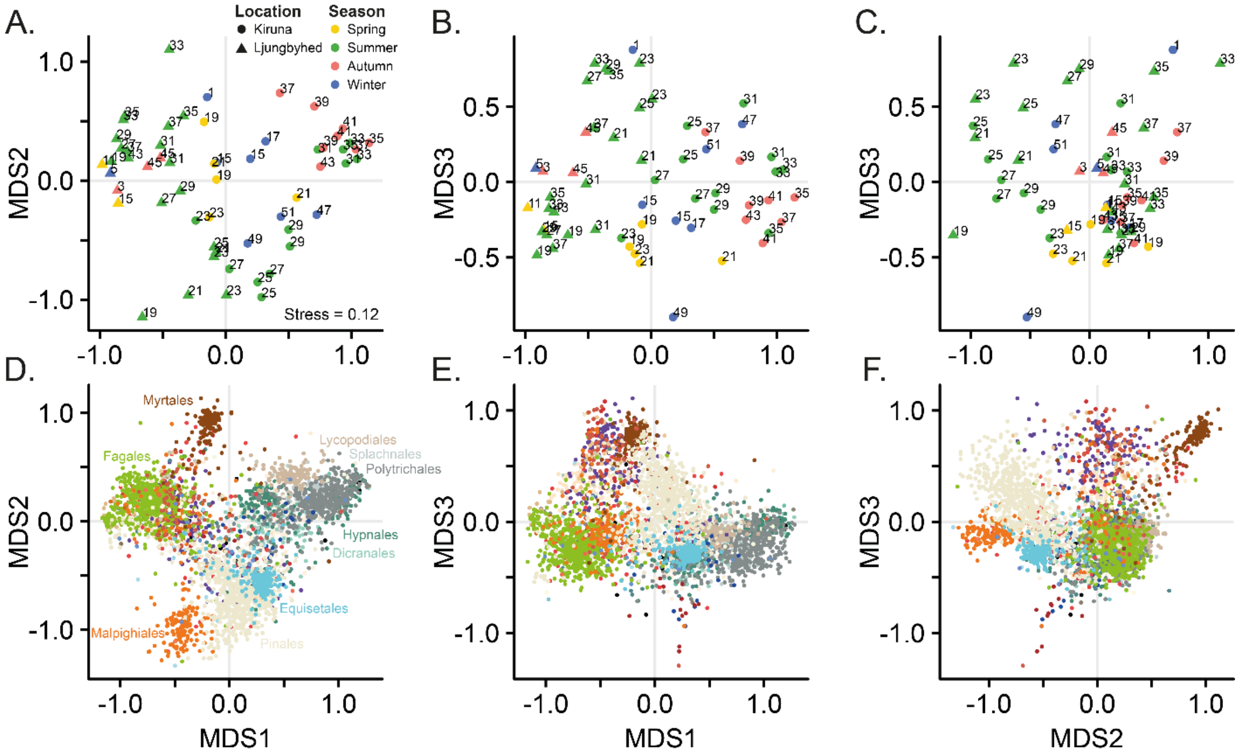

Figure S3. Non-metric Multidimensional Scaling (NMDS) ordination based on Bray-Curtis dissimilarities of Hellinger transformed sequence counts assigned to plants (*rbcL*). **(A, B, C)** Sample scores are shown for the first (MDS1), second (MDS2) and third (MDS3) dimension. The samples are labelled by week number. **(D, E, F)** The corresponding OTU scores for the three dimensions are colored according to taxonomic identity (order). For clarity, only the 20% most abundant OTUs are displayed. Color keys are shown in Fig. S4.

Color codes for NMDS

Plants

- Plantae
- Plantae Bryophyta
- Plantae Bryophyta Andreaeopsida Andreaeles
- Plantae Bryophyta Bryopsida
- Plantae Bryophyta Bryopsida Bartramiales
- Plantae Bryophyta Bryopsida Bryales
- Plantae Bryophyta Bryopsida Buxbaumiales
- Plantae Bryophyta Bryopsida Dicranales
- Plantae Bryophyta Bryopsida Diphysciales
- Plantae Bryophyta Bryopsida Funariales
- Plantae Bryophyta Bryopsida Grimmiales
- Plantae Bryophyta Bryopsida Hypnales
- Plantae Bryophyta Bryopsida Hypnodendrales
- Plantae Bryophyta Bryopsida Orthotrichales
- Plantae Bryophyta Bryopsida Pottiales
- Plantae Bryophyta Bryopsida Splachnales
- Plantae Bryophyta Polytrichopsida Polytrichales
- Plantae Bryophyta Sphagnopsida Sphagnales
- Plantae Bryophyta Tetraphidopsida Tetraphidales
- Plantae Chlorophyta
- Plantae Lycopodiophyta Lycopodiopsida Isoetales
- Plantae Lycopodiophyta Lycopodiopsida Lycopodiales
- Plantae Magnoliophyta
- Plantae Magnoliophyta Liliopsida
- Plantae Magnoliophyta Liliopsida Alismatales
- Plantae Magnoliophyta Liliopsida Arecales
- Plantae Magnoliophyta Liliopsida Asparagales
- Plantae Magnoliophyta Liliopsida Dioscoreales
- Plantae Magnoliophyta Liliopsida Liliales
- Plantae Magnoliophyta Liliopsida Pandanales
- Plantae Magnoliophyta Liliopsida Poales
- Plantae Magnoliophyta Liliopsida Zingiberales
- Plantae Magnoliophyta Magnoliopsida
- Plantae Magnoliophyta Magnoliopsida Apiales
- Plantae Magnoliophyta Magnoliopsida Asterales
- Plantae Magnoliophyta Magnoliopsida Boraginales
- Plantae Magnoliophyta Magnoliopsida Brassicales
- Plantae Magnoliophyta Magnoliopsida Buxales
- Plantae Magnoliophyta Magnoliopsida Caryophyllales
- Plantae Magnoliophyta Magnoliopsida Celastrales
- Plantae Magnoliophyta Magnoliopsida Cornales
- Plantae Magnoliophyta Magnoliopsida Crossosomatales
- Plantae Magnoliophyta Magnoliopsida Cucurbitales
- Plantae Magnoliophyta Magnoliopsida Dilleniales
- Plantae Magnoliophyta Magnoliopsida Dipsacales
- Plantae Magnoliophyta Magnoliopsida Ericales
- Plantae Magnoliophyta Magnoliopsida Fabales
- Plantae Magnoliophyta Magnoliopsida Fagales
- Plantae Magnoliophyta Magnoliopsida Gentianales
- Plantae Magnoliophyta Magnoliopsida Geraniales
- Plantae Magnoliophyta Magnoliopsida Gunnerales
- Plantae Magnoliophyta Magnoliopsida Lamiales
- Plantae Magnoliophyta Magnoliopsida Magnoliales
- Plantae Magnoliophyta Magnoliopsida Malpighiales
- Plantae Magnoliophyta Magnoliopsida Malvales
- Plantae Magnoliophyta Magnoliopsida Myrtales
- Plantae Magnoliophyta Magnoliopsida Oxalidales
- Plantae Magnoliophyta Magnoliopsida Proteales
- Plantae Magnoliophyta Magnoliopsida Ranunculales
- Plantae Magnoliophyta Magnoliopsida Rosales
- Plantae Magnoliophyta Magnoliopsida Sapindales
- Plantae Magnoliophyta Magnoliopsida Saxifragales
- Plantae Magnoliophyta Magnoliopsida Solanales
- Plantae Magnoliophyta unspecified unspecified
- Plantae Pinophyta Pinidae Pinales
- Plantae Pteridophyta Polypodiidae Polypodiales
- Plantae Pteridophyta Psilotopsida Ophioglossales
- Plantae Pteridophyta Pteridopsida Equisetales

Bacteria

- Bacteria [Thermi] Deinococci Deinococcales
- Bacteria Acidobacteria [Chloracidobacteria] RB41
- Bacteria Acidobacteria Acidobacteria-6 iii1-15
- Bacteria Acidobacteria Acidobacteriia Acidobacteriales
- Bacteria Acidobacteria Solibacteres Solibacterales
- Bacteria Actinobacteria Acidimicrobiia Acidimicrobiales
- Bacteria Actinobacteria Actinobacteria Actinomycetales
- Bacteria Actinobacteria Actinobacteria Bifidobacteriales
- Bacteria Actinobacteria Coriobacteriia Coriobacteriales
- Bacteria Actinobacteria MB-A2-108 0319-7L14
- Bacteria Actinobacteria Nitriliruptoria Nitriliruptorales
- Bacteria Actinobacteria Rubrobacteria Rubrobacterales
- Bacteria Actinobacteria Thermoleophilia Gaiellales
- Bacteria Actinobacteria Thermoleophilia Solirubrobacterales
- Bacteria Armatimonadetes [Fimbriimonadia] [Fimbriimonadales]
- Bacteria Armatimonadetes Armatimonadia FW68
- Bacteria Armatimonadetes Chthonomonadetes Chthonomonadales
- Bacteria Bacteroidetes [Saprospirae] [Saprospirales]
- Bacteria Bacteroidetes Bacteroidia Bacteroidales
- Bacteria Bacteroidetes Cytophagia Cytophagales
- Bacteria Bacteroidetes Flavobacteriia Flavobacteriales
- Bacteria Bacteroidetes Sphingobacteriia Sphingobacteriales
- Bacteria Chloroflexi Ellin6529
- Bacteria Chloroflexi Gitt-GS-136
- Bacteria Chloroflexi Ktedonobacteria Ktedonobacterales
- Bacteria Chloroflexi Ktedonobacteria Thermogemmatisporales
- Bacteria Chloroflexi Thermomicrobia JG30-KF-CM45
- Bacteria Chloroflexi TK17
- Bacteria Cyanobacteria Nostocophycideae Nostocales
- Bacteria Cyanobacteria Nostocophycideae Stigonematales
- Bacteria Cyanobacteria Oscillatoriophycideae Chroococcales
- Bacteria Cyanobacteria Oscillatoriophycideae Oscillatoriales
- Bacteria Cyanobacteria Synechococcophycideae Pseudanabaenales
- Bacteria Cyanobacteria Synechococcophycideae Synechococcales
- Bacteria FBP
- Bacteria Firmicutes Bacilli
- Bacteria Firmicutes Bacilli Bacillales
- Bacteria Firmicutes Bacilli Gemellales
- Bacteria Firmicutes Bacilli Haloplasmatales
- Bacteria Firmicutes Bacilli Lactobacillales
- Bacteria Firmicutes Bacilli Turicibacterales
- Bacteria Firmicutes Clostridia
- Bacteria Firmicutes Clostridia BSA2B-08
- Bacteria Firmicutes Clostridia Clostridiales
- Bacteria Firmicutes Clostridia MBA08
- Bacteria Firmicutes Clostridia Natranaerobiales
- Bacteria Firmicutes Clostridia OPB54
- Bacteria Firmicutes Clostridia SHA-98
- Bacteria Firmicutes Clostridia Thermoanaerobacterales
- Bacteria Firmicutes Erysipelotrichi Erysipelotrichales
- Bacteria Fusobacteria Fusobacteriia Fusobacteriales
- Bacteria Gemmatimonadetes Gemm-1
- Bacteria Gemmatimonadetes Gemmatimonadetes Gemmatimonadales
- Bacteria Planctomycetes Phycisphaerae WD2101
- Bacteria Planctomycetes Planctomycetia Gemmatales
- Bacteria Planctomycetes Planctomycetia Pirellulales
- Bacteria Proteobacteria
- Bacteria Proteobacteria Alphaproteobacteria
- Bacteria Proteobacteria Alphaproteobacteria Caulobacterales
- Bacteria Proteobacteria Alphaproteobacteria Rhizobiales
- Bacteria Proteobacteria Alphaproteobacteria Rhodobacterales
- Bacteria Proteobacteria Alphaproteobacteria Rhodospirillales
- Bacteria Proteobacteria Alphaproteobacteria Rickettsiales
- Bacteria Proteobacteria Alphaproteobacteria Sphingomonadales
- Bacteria Proteobacteria Betaproteobacteria Burkholderiales
- Bacteria Proteobacteria Betaproteobacteria Ellin6067
- Bacteria Proteobacteria Betaproteobacteria Methylophilales
- Bacteria Proteobacteria Betaproteobacteria Neisseriales
- Bacteria Proteobacteria Betaproteobacteria Rhodocyclales
- Bacteria Proteobacteria Deltaproteobacteria Bdellovibrionales
- Bacteria Proteobacteria Deltaproteobacteria Myxococcales
- Bacteria Proteobacteria Deltaproteobacteria Spirobacillales
- Bacteria Proteobacteria Gammaproteobacteria
- Bacteria Proteobacteria Gammaproteobacteria Alteromonadales
- Bacteria Proteobacteria Gammaproteobacteria Cardiobacteriales
- Bacteria Proteobacteria Gammaproteobacteria Enterobacteriales
- Bacteria Proteobacteria Gammaproteobacteria Legionellales
- Bacteria Proteobacteria Gammaproteobacteria Oceanospirillales
- Bacteria Proteobacteria Gammaproteobacteria Pasteurellales
- Bacteria Proteobacteria Gammaproteobacteria Pseudomonadales
- Bacteria Proteobacteria Gammaproteobacteria Thiotrichales
- Bacteria Proteobacteria Gammaproteobacteria Vibrionales
- Bacteria Proteobacteria Gammaproteobacteria Xanthomonadales
- Bacteria Tenericutes Mollicutes Entomoplasmatales
- Bacteria Tenericutes Mollicutes RF39
- Bacteria Verrucomicrobia [Spartobacteria] [Chthoniobacterales]
- Bacteria Verrucomicrobia Opitutae Puniceicoccales
- Bacteria Verrucomicrobia Verrucomicrobiae Verrucomicrobiales
- Bacteria WPS-2

Fungi

- Fungi Ascomycota Archaeorhizomycetes Archaeorhizomycetales
- Fungi Ascomycota Dothideomycetes Botryosphaeriales
- Fungi Ascomycota Dothideomycetes Capnodiales
- Fungi Ascomycota Dothideomycetes Dothideales
- Fungi Ascomycota Dothideomycetes Incertae sedis
- Fungi Ascomycota Dothideomycetes Myriangiales
- Fungi Ascomycota Dothideomycetes Mytilinidiales
- Fungi Ascomycota Dothideomycetes Pleosporales
- Fungi Ascomycota Dothideomycetes Tubeufiales
- Fungi Ascomycota Dothideomycetes unidentified
- Fungi Ascomycota Dothideomycetes Venturiales
- Fungi Ascomycota Eurotiomycetes Chaetothyriales
- Fungi Ascomycota Eurotiomycetes Eurotiales
- Fungi Ascomycota Eurotiomycetes Incertae sedis
- Fungi Ascomycota Eurotiomycetes Mycocaliciales
- Fungi Ascomycota Eurotiomycetes Onygenales
- Fungi Ascomycota Eurotiomycetes Pyrenulales
- Fungi Ascomycota Eurotiomycetes Verrucariales
- Fungi Ascomycota Geoglossomycetes Geoglossales
- Fungi Ascomycota Incertae sedis Incertae sedis
- Fungi Ascomycota Lecanoromycetes Acarosporales
- Fungi Ascomycota Lecanoromycetes Agyriales
- Fungi Ascomycota Lecanoromycetes Candelariales
- Fungi Ascomycota Lecanoromycetes Incertae sedis
- Fungi Ascomycota Lecanoromycetes Lecanorales
- Fungi Ascomycota Lecanoromycetes Lecideales
- Fungi Ascomycota Lecanoromycetes Peltigerales
- Fungi Ascomycota Lecanoromycetes Pertusariales
- Fungi Ascomycota Lecanoromycetes Teloschistales
- Fungi Ascomycota Lecanoromycetes Umbilicariales
- Fungi Ascomycota Lecanoromycetes unidentified
- Fungi Ascomycota Leotiomycetes Erysiphales
- Fungi Ascomycota Leotiomycetes Helotiales
- Fungi Ascomycota Leotiomycetes Incertae sedis
- Fungi Ascomycota Leotiomycetes Leotiales
- Fungi Ascomycota Leotiomycetes Rhytismatales
- Fungi Ascomycota Leotiomycetes unidentified
- Fungi Ascomycota Orbiliomycetes Orbiliales
- Fungi Ascomycota Pezizomycetes Pezizales
- Fungi Ascomycota Saccharomycetes Saccharomycetales
- Fungi Ascomycota Sordariomycetes Boliniales
- Fungi Ascomycota Sordariomycetes Calosphaeriales
- Fungi Ascomycota Sordariomycetes Chaetosphaeriales
- Fungi Ascomycota Sordariomycetes Coniochaetales
- Fungi Ascomycota Sordariomycetes Diaporthales
- Fungi Ascomycota Sordariomycetes Glomerellales
- Fungi Ascomycota Sordariomycetes Hypocreales
- Fungi Ascomycota Sordariomycetes Incertae sedis
- Fungi Ascomycota Sordariomycetes Lulworthiales
- Fungi Ascomycota Sordariomycetes Microascales
- Fungi Ascomycota Sordariomycetes Ophiostomatales
- Fungi Ascomycota Sordariomycetes Sordariales
- Fungi Ascomycota Sordariomycetes unidentified
- Fungi Ascomycota Sordariomycetes Xylariales
- Fungi Ascomycota Taphrinomycetes Taphrinales
- Fungi Ascomycota unidentified unidentified
- Fungi Basidiomycota Agaricomycetes Agaricales
- Fungi Basidiomycota Agaricomycetes Atheliales
- Fungi Basidiomycota Agaricomycetes Auriculariales
- Fungi Basidiomycota Agaricomycetes Boletales
- Fungi Basidiomycota Agaricomycetes Cantharellales
- Fungi Basidiomycota Agaricomycetes Corticiales
- Fungi Basidiomycota Agaricomycetes Gloeophyllales
- Fungi Basidiomycota Agaricomycetes Hymenochaetales
- Fungi Basidiomycota Agaricomycetes Incertae sedis
- Fungi Basidiomycota Agaricomycetes Phallales
- Fungi Basidiomycota Agaricomycetes Polyporales
- Fungi Basidiomycota Agaricomycetes Russulales
- Fungi Basidiomycota Agaricomycetes Sebacinales
- Fungi Basidiomycota Agaricomycetes Thelephorales
- Fungi Basidiomycota Agaricomycetes Trechisporales
- Fungi Basidiomycota Agaricomycetes unidentified
- Fungi Basidiomycota Agaricostilbomycetes Agaricostilbales
- Fungi Basidiomycota Agaricostilbomycetes unidentified
- Fungi Basidiomycota Cystobasidiomycetes Erythrobasidiales
- Fungi Basidiomycota Exobasidiomycetes Incertae sedis
- Fungi Basidiomycota Incertae sedis Malasseziales
- Fungi Basidiomycota Microbotryomycetes Leucosporidiales
- Fungi Basidiomycota Microbotryomycetes Sporidiobolales
- Fungi Basidiomycota Microbotryomycetes unidentified
- Fungi Basidiomycota Pucciniomycetes Helicobasidiales
- Fungi Basidiomycota Pucciniomycetes Platygloeales
- Fungi Basidiomycota Pucciniomycetes Pucciniales
- Fungi Basidiomycota Tremellomycetes Cystofilobasidiales
- Fungi Basidiomycota Tremellomycetes Filobasidiales
- Fungi Basidiomycota Tremellomycetes Tremellales
- Fungi Basidiomycota Tremellomycetes Trichosporonales
- Fungi Basidiomycota Tremellomycetes unidentified
- Fungi Basidiomycota unidentified unidentified
- Fungi Basidiomycota Wallemiomycetes Geminibasidiales
- Fungi Basidiomycota Wallemiomycetes unidentified
- Fungi Basidiomycota Wallemiomycetes Wallemiales
- Fungi Chytridiomycota Chytridiomycetes Spizellomycetales
- Fungi unidentified unidentified unidentified
- Fungi Zygomycota Incertae sedis Mortierellales
- Fungi Zygomycota Incertae sedis Mucorales

Figure S4. Extended legend for the NMDS OTU score plots in Fig. 1 and FigS3.

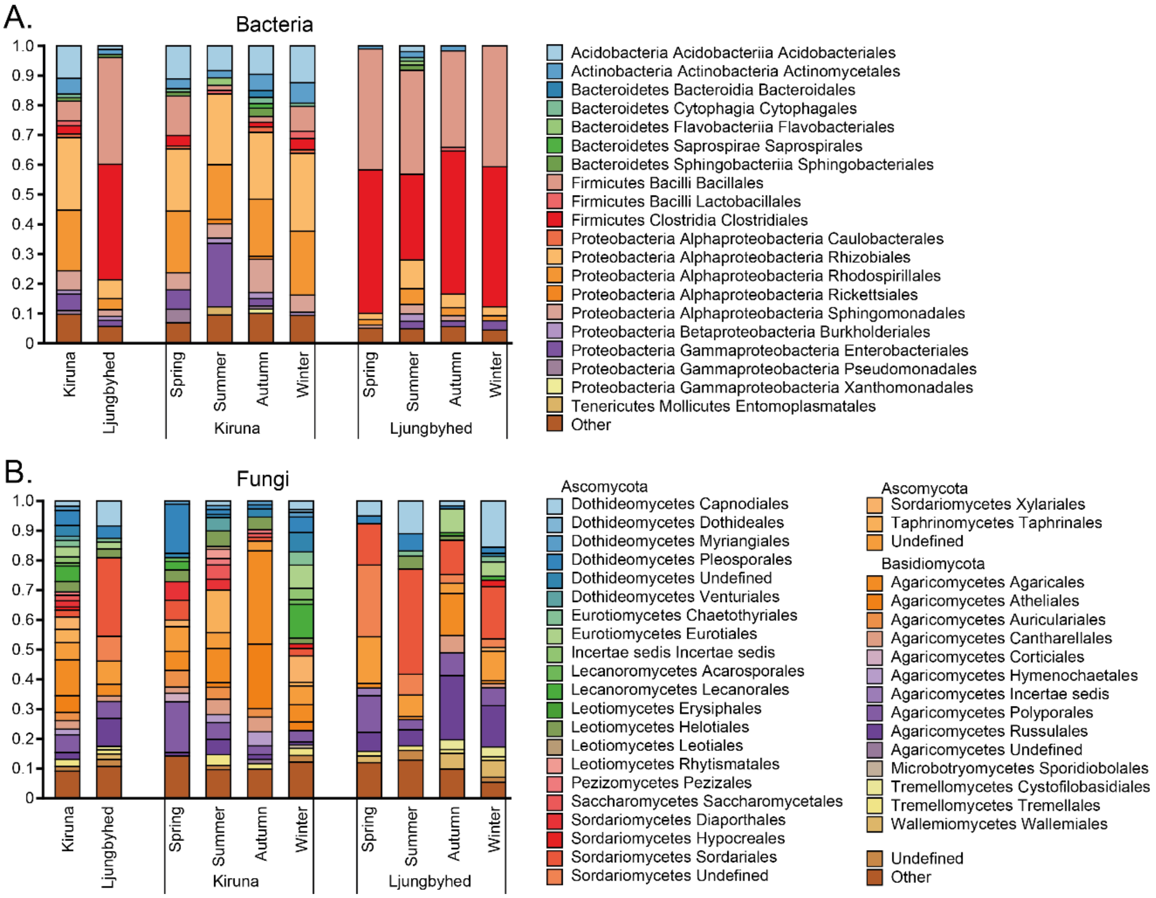

Figure S5. Taxonomic summary at order level for **(A)** bacteria and **(B)** fungi in Kiruna and Ljungbyhed across the sampling period and seasons. Orders below 1% of the total sequence abundance are grouped as "Other".

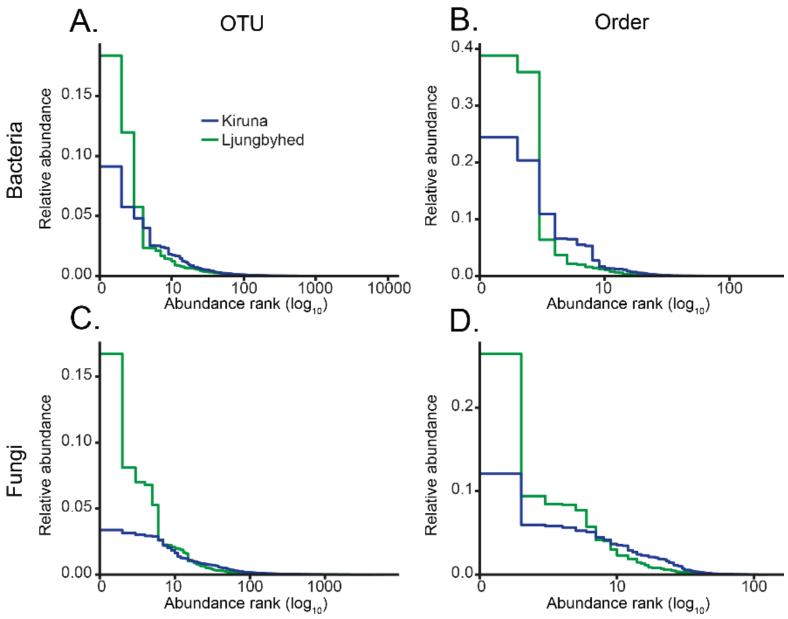

Figure S6. Ranked relative abundance for **(A)** bacterial OTUs, **(B)** bacterial orders, **(C)** fungal OTUs and **(D)** fungal orders in Kiruna (blue) and Ljungbyhed (green).

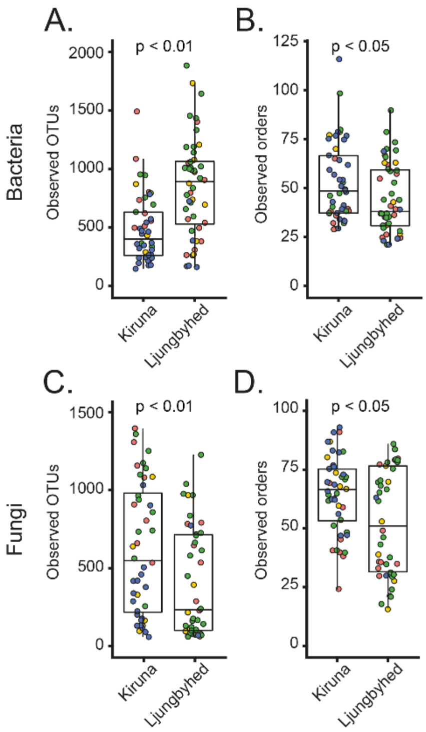

Figure S7. Distribution of observed number of **(A)** bacterial OTUs, **(B)** bacterial orders, **(C)** fungal OTUs and **(D)** fungal orders in Kiruna and Ljungbyhed. Jitter points represents each individual week, colored by season; spring (yellow), summer (green), autumn (red) and winter (blue). The p-values were calculated using the Wilcoxon rank-sum test.

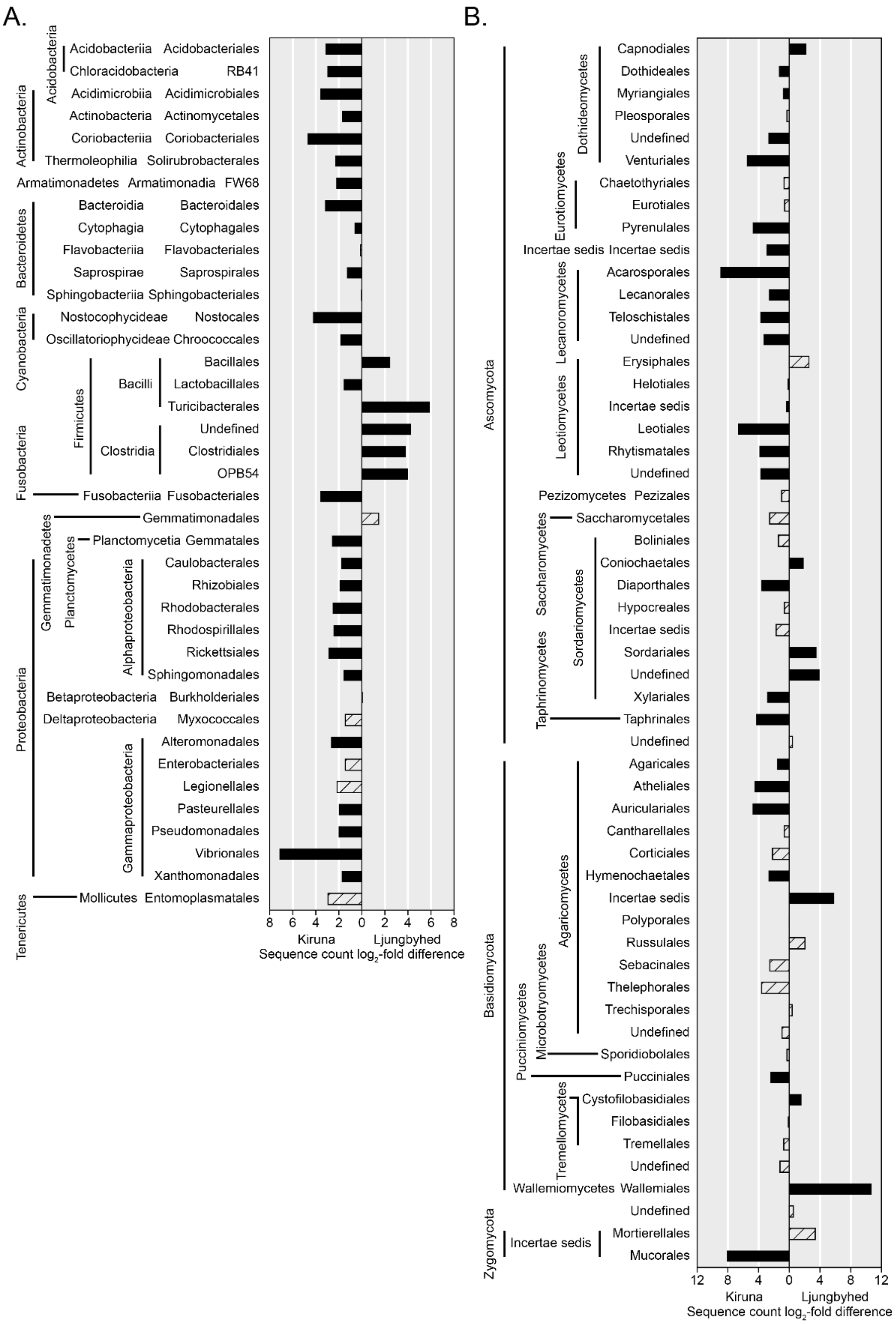

Figure S8. Geographic comparison of **(A)** bacterial and **(B)** fungal order composition between Kiruna and Ljungbyhed. Bars represent the log_2-fold difference in sequence abundance for each individual order. Significant differences are illustrated by filled bars (Wilcoxon rank-sum test, $p < 0.01$). Values are adjusted for the differences in total sequence abundance between the two locations. Orders with a sequence abundance below 0.1% of the total sequence abundance are not shown.

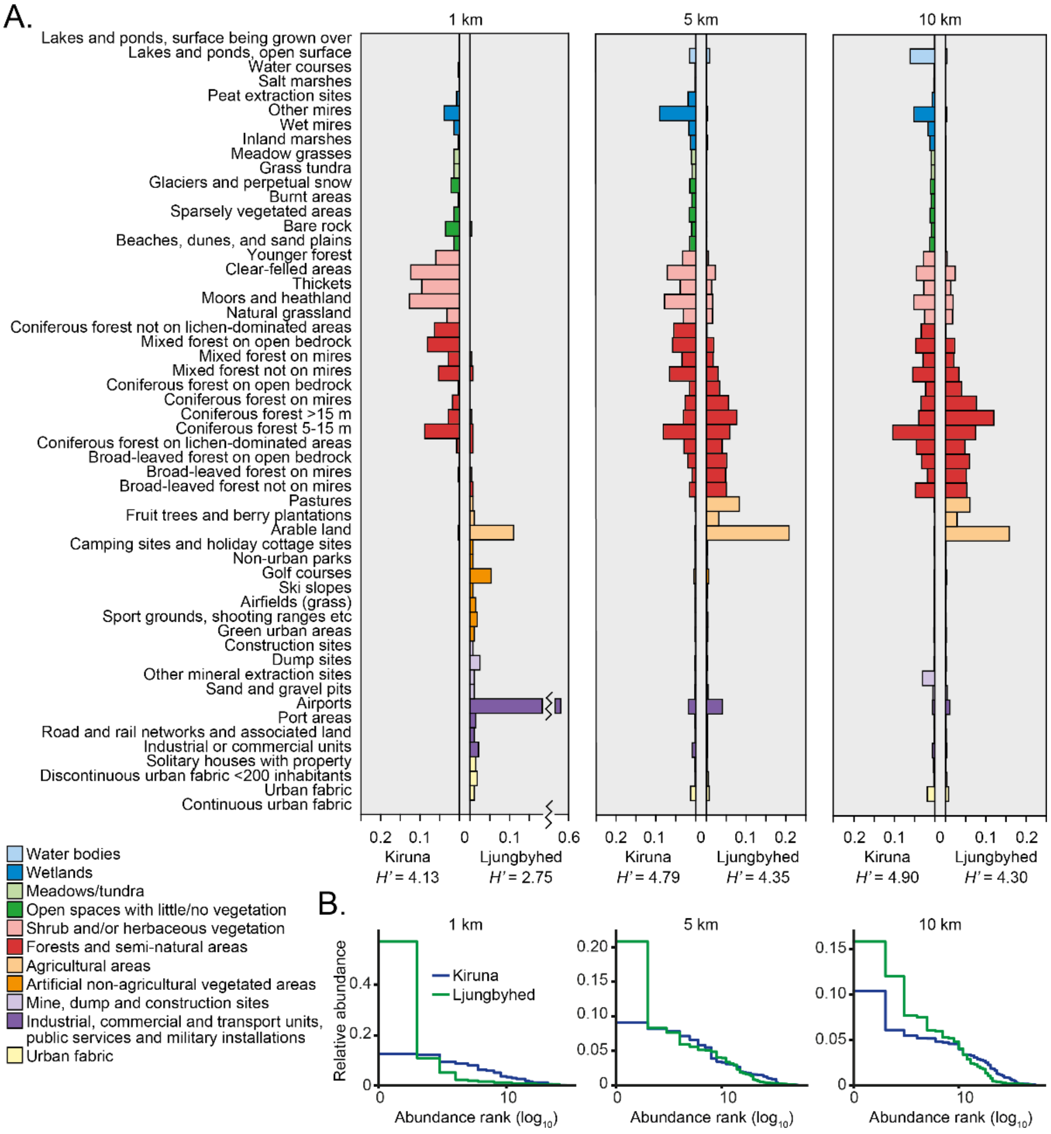

Figure S9. (A) Proportion of land cover types surrounding the air filter stations in Kiruna and Ljungbyhed at a radius of 1 km, 5 km and 10 km. Land cover diversity within each area is shown as the Shannon entropy (H'). The bars are colored by land type category (see legend). **(B)** Ranked relative abundance (in $\log_{10}$ scale) of the land cover types within 1 km, 5 km and 10 km radius in Kiruna (blue) and Ljungbyhed (green).

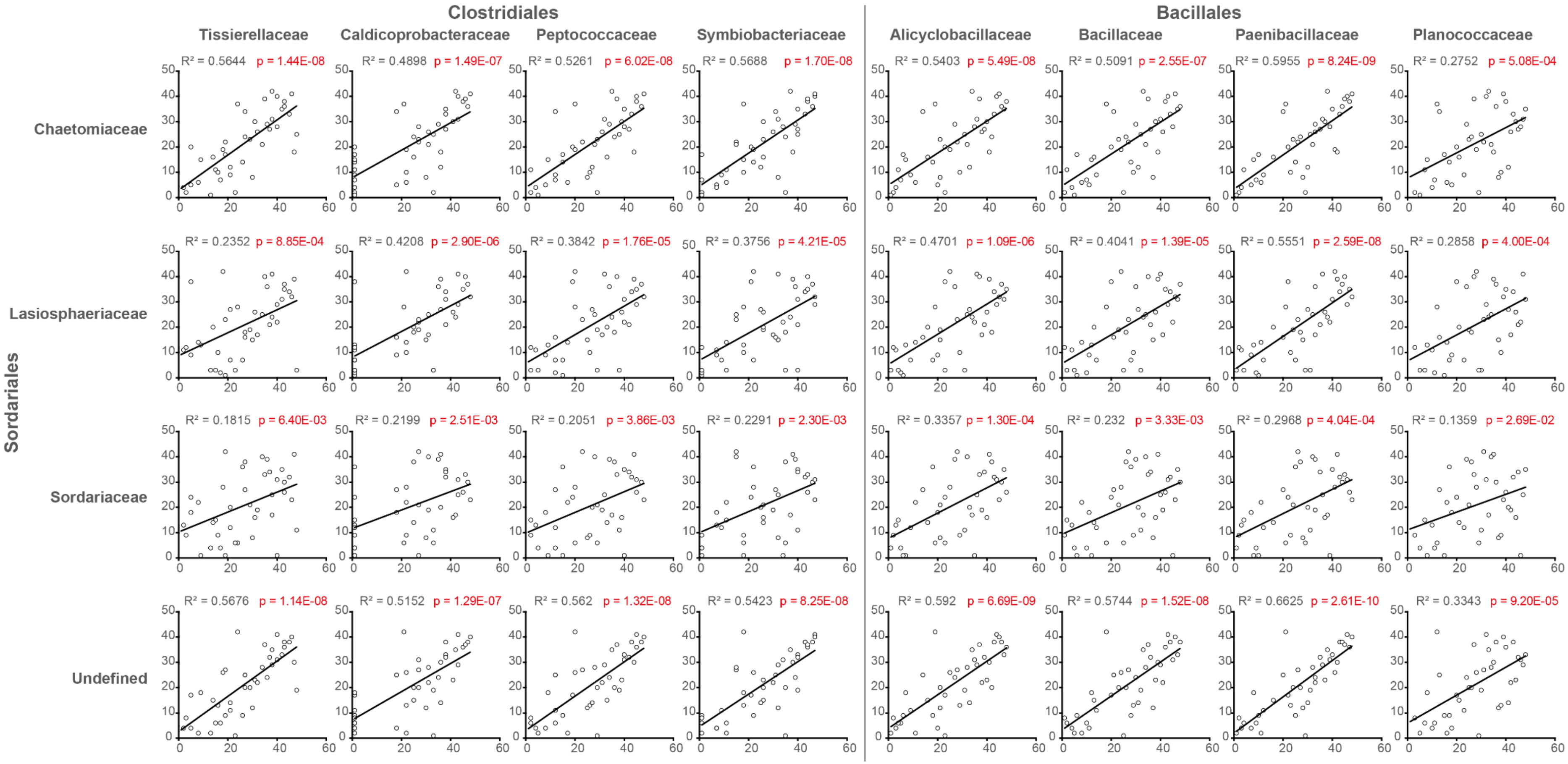

Figure S10. Ranked abundance scatter plots between families within the fungal order Sordariales and families within the bacterial orders Clostridiales and Bacillales. P-values below 0.05 are depicted in red.

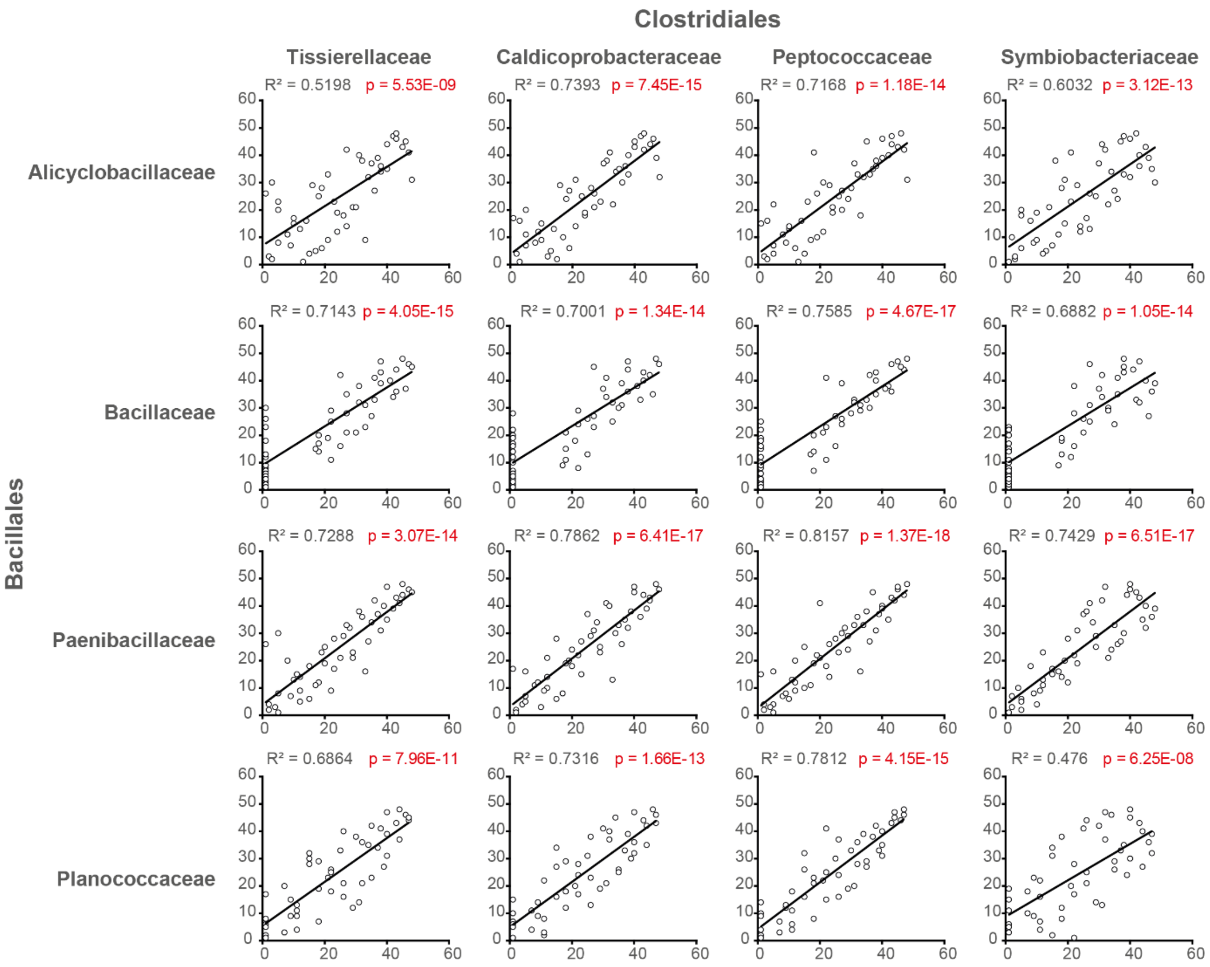

Figure S11. Ranked abundance scatter plots between families within the bacterial orders Clostridiales and Bacillales. P-values below 0.05 are depicted in red.

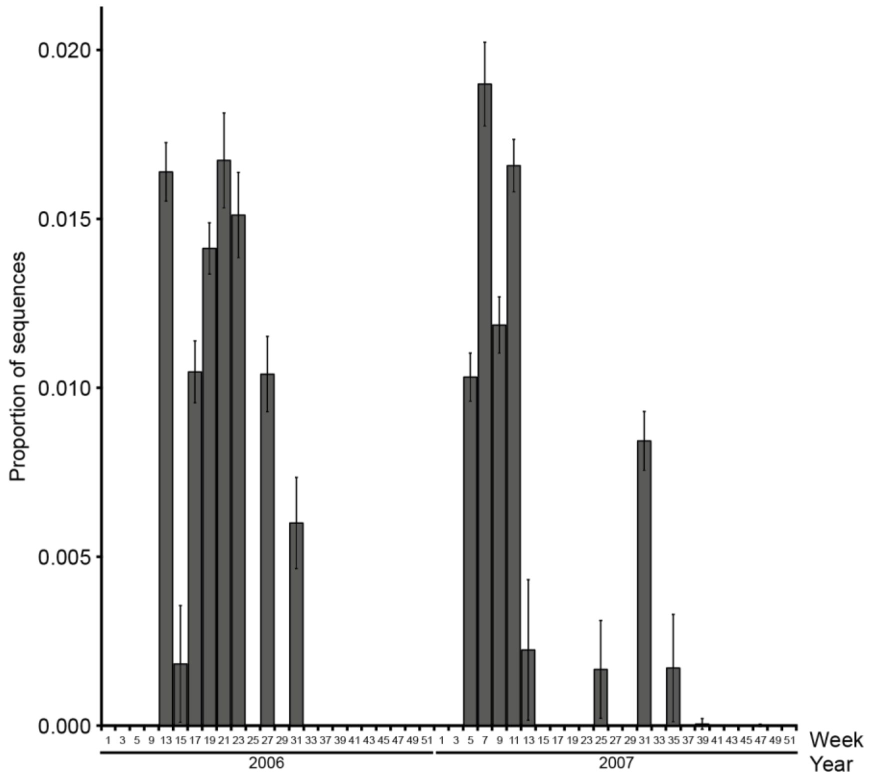

Figure S12. Proportion of sequences predicted to have a fecal origin, predicted as domestic bird and pig, across the two years in Ljungbyhed. Uncertainty of the proportions of sequences of fecal origin via the standard deviation is provided as error bars.

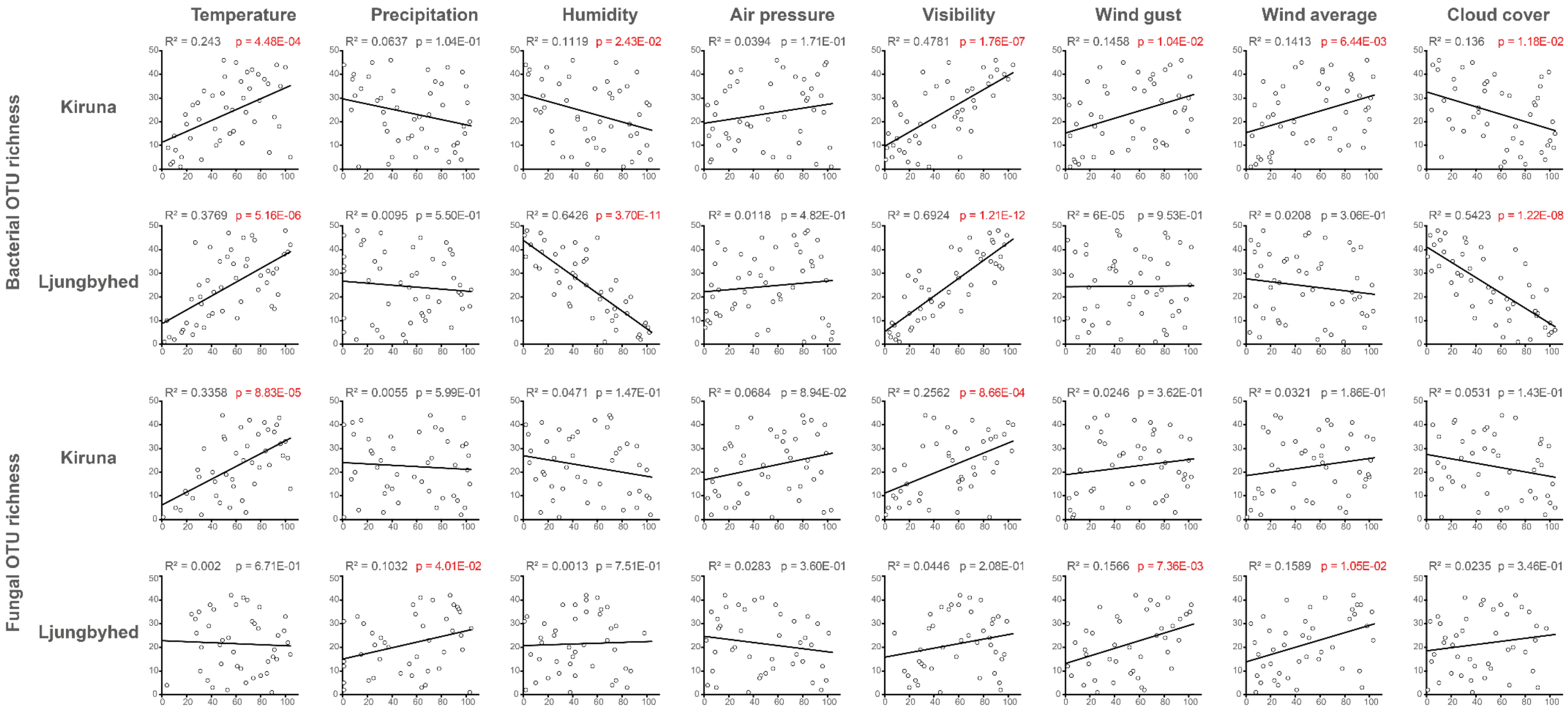

Figure S13. Relationship between ranked bacterial OTU and fungal OTU richness and ranked weather parameters. P-values were calculated using Spearman’s correlation. P-values below 0.05 are depicted in red.

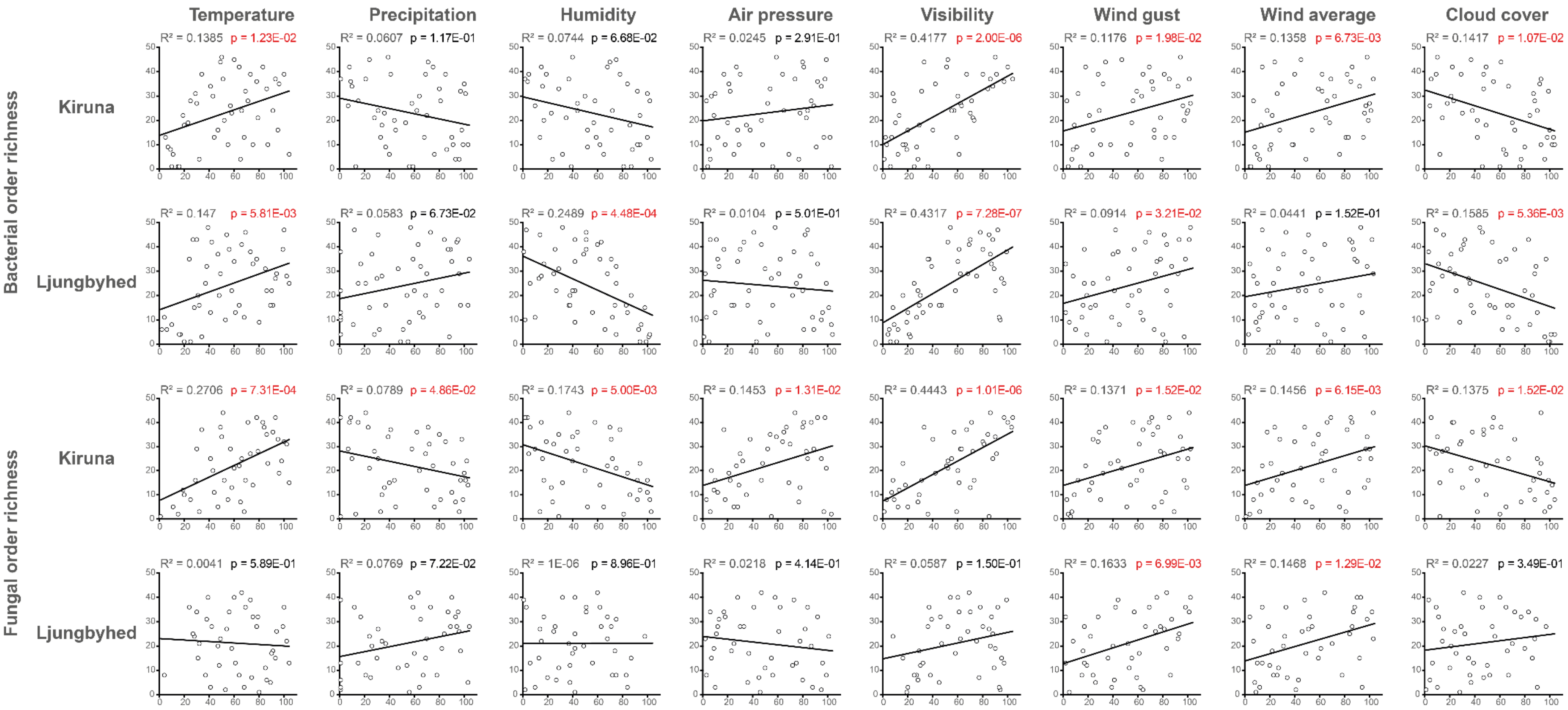

Figure S14. Relationship between ranked bacterial order and fungal order richness and ranked weather parameters. P-values were calculated using Spearman's correlation. P-values below 0.05 are depicted in red.

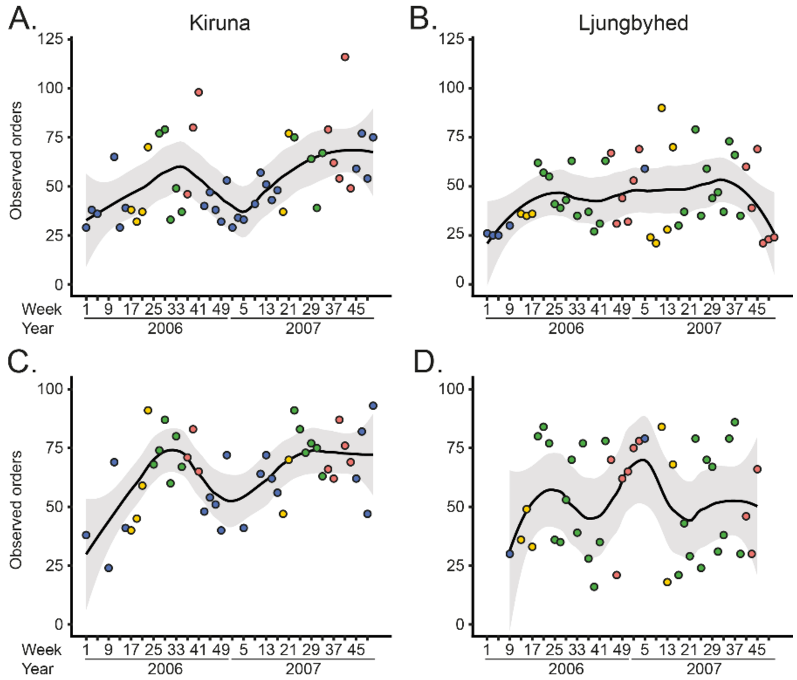

Figure S15. The weekly observed number of bacterial orders in **(A)** Kiruna and **(B)** Ljungbyhed, and the weekly observed number of fungal orders in **(C)** Kiruna and **(D)** Ljungbyhed. Weeks are colored by season (spring; yellow, summer; green, autumn; red and winter; blue). A local regression (LOESS) curve is fitted to the observations to display seasonal trends (black line). The standard error of the LOESS curve is depicted in grey.

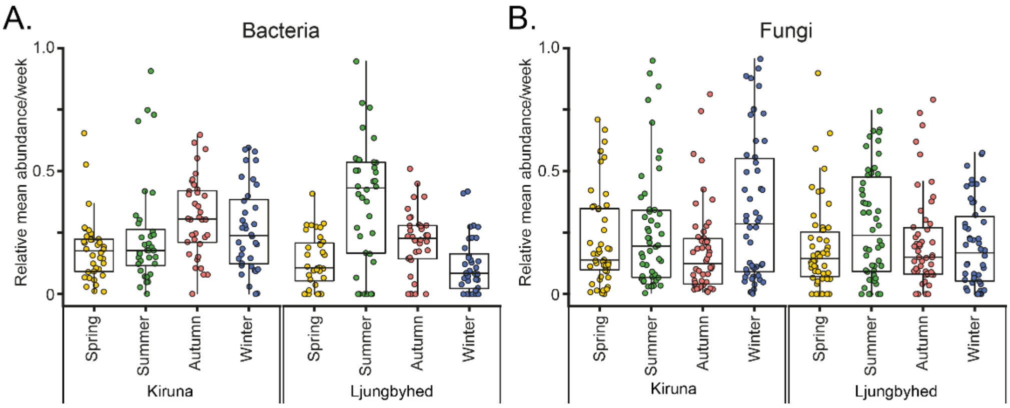

Figure S16. Distribution of the relative mean order abundance per week by season for **(A)** fungal and **(B)** bacterial orders in Kiruna and Ljungbyhed.

References

1. Walters W, Hyde ER, Berg-Lyons D, Ackermann G, Humphrey G, Parada A, Gilbert JA, Jansson JK, Caporaso JG, Fuhrman JA, Apprill A and Knight R (2016) Improved Bacterial 16S rRNA Gene (V4 and V4-5) and Fungal Internal Transcribed Spacer Marker Gene Primers for Microbial Community Surveys. mSystems 1:
2. Parada AE, Needham DM and Fuhrman JA (2016) Every base matters: assessing small subunit rRNA primers for marine microbiomes with mock communities, time series and global field samples. Environ Microbiol 18:1403-14
3. Apprill A, McNally S, Parsons R and Weber L (2015) Minor revision to V4 region SSU rRNA 806R gene primer greatly increases detection of SAR11 bacterioplankton. Aquat Microb Ecol 75:129-137
4. Gardes M and Bruns TD (1993) ITS primers with enhanced specificity for basidiomycetes--application to the identification of mycorrhizae and rusts. Mol Ecol 2:113-8
5. White TJ, Bruns S, Lee S and Taylor J (1990) Amplification and direct sequencing of fungal ribosomal RNA genes for phylogenetics. In Innis, MA, Gelfand, DH, Sninsky, JJ and White, TJ (Eds.). PCR - Protocols and Applications - A Laboratory Manual. 1st ed:315-322. New York, NY. Academic Press
6. Hofreiter M, Poinar HN, Spaulding WG, Bauer K, Martin PS, Possnert G and Paabo S (2000) A molecular analysis of ground sloth diet through the last glaciation. Mol Ecol 9:1975-84
